# DAMP Laden Extracellular Vesicles From the Airways of Patients With Severe SARS‐CoV‐2 Respiratory Infection Compromise Inflammation and Cellular Metabolism

**DOI:** 10.1002/jev2.70288

**Published:** 2026-05-28

**Authors:** April Rees, Oliver Richards, Molly E Raikes, Megan Chambers, Sophie G Reed, Ceri Battle, Hannah Toghill, Luke Newey, Tyler J Joseph, Haiyan An, Hui Zhang, Iain Perry, Jason Webber, Nicholas Jones, Catherine A Thornton

**Affiliations:** ^1^ Institute of Life Science Swansea University Medical School Swansea Wales UK; ^2^ Physiotherapy Morriston Hospital Swansea Bay University Health Board Swansea Wales UK; ^3^ Cryo‐EM Facility School of Biological & Behavioural Sciences Queen Mary University of London, England London UK

**Keywords:** aspirates, blood, extracellular vesicles, macrophages, monocytes, SARS‐CoV‐2

## Abstract

Highly inflammatory mononuclear phagocytes (MNPs) cause tissue damage across the respiratory tract and other organs in response to infections such as SARS‐CoV‐2. Extracellular vesicles (EVs) can metabolically and phenotypically reprogram target cells, suggesting a potential role in COVID‐19 pathology. We hypothesised that *EVs drive inflammatory changes in COVID‐19* and investigated their cargo and effects on MNPs. EVs and MNPs were isolated from matched peripheral blood and airways of ventilated patients with and without severe COVID‐19, alongside healthy volunteer blood samples. Characterisation was performed using flow cytometry, microscopy, transcriptomics, PCR, proteomics, mass spectrometry, and bioenergetic profiling. We found that airway EVs from severe COVID‐19 patients carried altered cargo, notably reduced miRNAs and enriched mitochondrial DNA and ATP, a difference absents in the circulation. These DAMP‐rich EVs impaired healthy MNP function, suppressing cytokine production (e.g., IL‐6, IFNα2), and inflammatory programs identified in the monocyte proteomic profile, while also disrupting oxidative phosphorylation—features matching airway MNPs in severe disease. Interaction of COVID‐19 airway EVs with monocytes was diminished and these EVs had altered phosphatidylcholine/ lysophosphatidylcholine content. Our findings reveal a distinct EV cargo that reprograms immune metabolism, identifying a novel immunomodulatory mechanism exploited by SARS‐CoV‐2.

## Graphical abstract

Extracellular vesicles from severe COVID‐19 airways carry depleted miRNAs and elevated DAMPs, reprogramming monocytes toward senescence, impaired metabolism, and predominantly reduced cytokine responses. However, they drive excess IL‐8 production, amplifying neutrophil recruitment and dysfunctional inflammation across the respiratory tract. Made with BioRender.

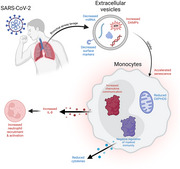

**Abbreviations**
BAF: bronchial aspirate fluid
MNCs: mononuclear cells
EVs: extracellular vesicles
MNPs: mononuclear phagocytes
COVID‐19: coronavirus disease 19
SARS‐CoV‐2: severe acute respiratory syndrome coronavirus 2.

## 1 Introduction

Hyperinflammation is a feature of severe COVID‐19 underpinned by dysfunction of mononuclear phagocytes (MNPs) (Junqueira et al. 2022). Highly inflammatory blood MNPs traffic to the airways and supplant reparative local MNPs, causing pulmonary damage, concomitant with migration to the heart, kidneys, and other tissues, provoking localised tissue damage and dysfunction (Junqueira et al. 2022). Continuous elevation of cytokines contributes to auto‐amplification of inflammation and increased vascular permeability, thrombosis, organ failure and subsequently, death. The mechanistic determinants of hyperinflammation are unknown but intracellular events, such as cellular metabolic adaptation which provide energy and support the production of biosynthetic intermediates, along with inflammasome activation, might drive the hyperactivated MNP phenotype (O'Neill and Pearce 2016, Sefik et al. 2022).

Immunometabolic modulation of immune cells occurs in multiple viral infections (Rao et al. 2019). HIV, for example, hijacks the glycolytic pathway of MNPs to support viral replication resulting in functional glucose restriction inhibiting the ability of MNPs to clear the virus (Lin et al. 2022, Palmer et al. 2016). Human epithelial cells and monocytes infected in vitro with SARS‐CoV‐2 modulate translational and metabolic pathways linked to virus replication and cellular responses such as cytokine production, with the cells becoming highly glycolytic and contributing to cytokine storm (Bojkova et al. 2020, Codo et al. 2020). To date, the key drivers of these metabolic perturbations remain unknown.

Extracellular vesicles (EVs) have been shown to be essential mediators of intercellular communication, capable of influencing monocyte metabolism and effector function in other settings. Tumour‐derived (Gärtner et al. 2018) and cardiac‐derived adherent proliferating cell (Beez et al. 2019) EVs prime monocytes towards an activated and regulatory phenotype, respectively, whereas endothelial cell‐derived EVs suppress monocyte activation (Njock et al. 2015). EVs carry a heterogeneous cargo including macromolecules (proteins, lipids, enzymes) and genetic material (mRNA, miRNA, with possible inclusion of DNA and mtDNA remaining controversial) (Buzas 2023, Doyle and Wang 2019). The cellular origin of the EVs, and mechanism of biogenesis, can influence cargo composition, whereby EVs from different organs or cell types affect target cells uniquely (Zhang et al. 2019). The profile of circulating EVs remains distinct several weeks post‐infection with SARS‐CoV‐2, particularly the protein content, suggestive of endothelial, platelet and immune cell‐derived EVs (Barion et al. 2024). Co‐culture of healthy T cells with circulating EVs from mild SARS‐CoV‐2 infection reduces metabolism and cytokine production, whereas circulating EVs from patients with severe infection increased glucose metabolism and effector cytokines (George et al. 2023). To date, there are no studies of EVs from the SARS‐CoV‐2 infected airways. Given that circulating EVs during severe SARS‐Cov‐2 infection promote inflammation (Fujita et al. 2021, Krishnamachary et al. 2021) and other hallmarks of severe COVID‐19 such as coagulation (Balbi et al. 2021, Burrello et al. 2022), EVs within the airways could feasibly alter the inflammatory and metabolic status of key target cells such as MNPs.

We hypothesised that EVs are key drivers of the inflammatory changes that characterise COVID‐19. Here, we utilised matched samples of arterial blood and bronchial aspirate fluid (BAF) collected from the airways of ventilated patients with and without severe COVID‐19 during routine chest physiotherapy to investigate the circulating and tissue‐specific inflammatory environment and MNP functionality while establishing the contribution of EVs to inflammation both systemically and locally in the airways. Our study highlights that BAF‐derived EVs are intrinsically perturbed with SARS‐CoV‐2 infection with altered phenotype and cargo, which are associated with attenuation of the MNP response by affecting cell metabolism and thereby function. Responses shown in our *in vitro* model are recapitulated by phenotypic and functional characteristics shown for airway‐derived MNPs studied *ex vivo*. Taken together, this work demonstrates that EVs have unique functionality within the airways compared to the circulation and are part of a newly described viral immunomodulatory strategy that provides a novel therapeutic target.

## 2 Results

### 2.1 The Cellular and Molecular Content of Blood and BAF Is Altered With Severe SARS‐CoV‐2

**FIGURE 1:**
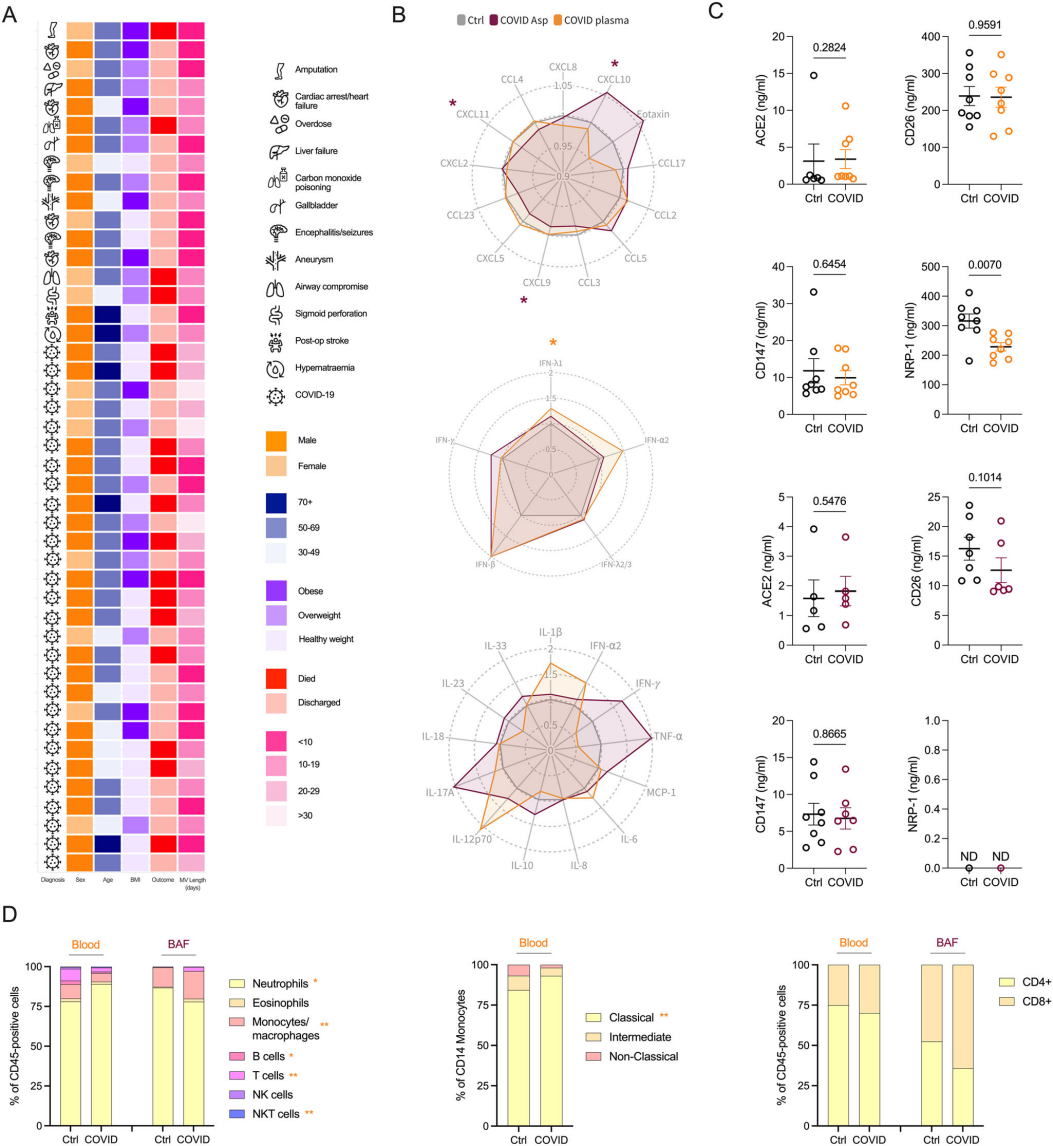
**Demographics of patients, and characterisation of blood and BAF. (A)** Diagnosis—reason for hospitalisation and intubation. Outcome—if the patient died or was discharged. BMI—healthy weight (< 25 kg/m^2^), overweight (25–29.9 kg/m^2^), obese (> 30 kg/m^2^); no participant was morbidly obese (> 40 kg/m^2^). medical ventilation (MV) duration (days). Orange is used to represent samples from blood, and maroon from aspirates. **(B)** LEGENDplex^TM^ panels for chemokines, interferon response, and cytokines were used to measure a total of 31 analytes for control (*n* = 10) and COVID‐19 infected (*n* = 20) patients in plasma (orange) and BAF (maroon) samples. For the radar plots, the mean concentration of control samples was used as the base to calculate the log of the COVID‐19 samples for plasma and for the aspirates. All control samples were then normalised to 1. **(C)** ELISAs were performed for ACE2, CD26, CD147 and NRP1 for control (*n* = 8) and COVID‐19 (*n* = 8) samples. Not all samples had detectable quantities of these analytes. **(D)** Leukocyte (CD45+) populations were analysed using the 8‐colour phenotyping panel for flow cytometry for control and COVID‐19 patients from whole blood (*n* = 10 and *n* = 13, respectively) and BAF (*n* = 6 and *n* = 4, respectively), where each cell population was compared in control versus COVID‐19 patients. All statistics were performed using a Mann–Whitney *t* test where *p* < 0.05 was deemed to be significant; represents *p <0.05, and **p<0.01.

We investigated intubated ICU patients with acute respiratory failure who were SARS‐CoV‐2–negative (controls, ventilated for non‐COVID‐19 conditions) or SARS‐CoV‐2–positive with severe COVID‐19 during the first wave of the pandemic; none of the controls had prior COVID‐19, before the introduction of vaccinations in the UK (Figure 1A). To provide baseline characterisation of study participants and evaluate the molecular content of plasma and BAF from intubated patients with and without SARS‐CoV‐2 infection, a multiplex approach was used to investigate various chemokines, cytokines, and interferons (Figure 1B; raw data shown in Figure S1). Here, levels of CXCL10 were increased significantly in the COVID‐19 airway samples, with decreases in CXCL9 and CXCL11 also observed. IFNλ was significantly elevated in plasma from COVID‐19 patients.

To better understand the local airways versus systemic environment we also measured soluble versions of receptors that facilitate SARS‐CoV‐2 entry into the cell including angiotensin converting enzyme 2 (ACE2) (Ni et al. 2020), dipeptidyl‐peptidase IV (CD26) (Vankadari and Wilce 2020), basigin (CD147) (Wang et al. 2020) and neuropilin‐1 (NRP‐1) (Cantuti‐Castelvetri et al. 2020) (Figure 1C). Here, NRP‐1 was significantly lower in the plasma from infected patients. We also investigated the leukocyte composition of the blood and BAF by utilising an 8‐colour flow cytometry panel (Figure 1D; Figure S2). Within the blood, neutrophils were elevated significantly in the COVID‐19 patients, and monocytes, particularly classical monocytes, B cells, T cells and NKT cells were all significantly reduced. There were no significant differences in the cellular composition of BAF from the two patient groups, however our analysis revealed that BAF is immunologically rich, with neutrophils, eosinophils, macrophages, B cells, T cells, NK cells and NKT cells all being present. Collectively, our data highlight that while the cellular composition of BAF did not differ significantly between groups, the soluble mediator milieu in both airways and blood was substantially altered in association with SARS‐CoV‐2 infection.

### 2.2 Peripheral and Airway MNPs Are Metabolically and Phenotypically Compromised in Patients With SARS‐CoV‐2 Infection

**FIGURE 2:**
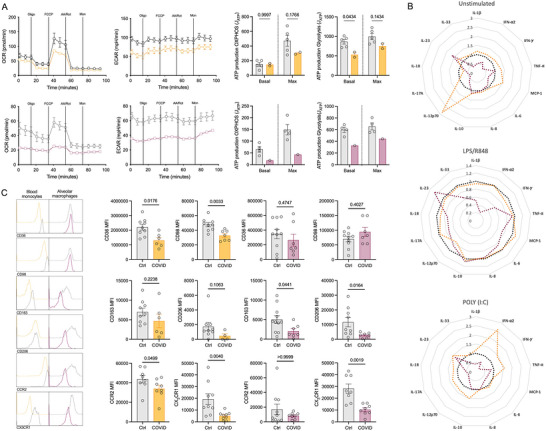
**Activity of MNPs with severe SARS‐CoV‐2 infection**. MNPs were isolated using magnetic microbeads or identified using flow cytometry from control (grey) and COVID‐19 patients using blood (orange) or BAF (maroon). **(A)** MNPs from blood (control *n* = 5, COVID‐19 *n* = 2) and BAF (control *n* = 4, COVID‐19 *n* = 1) were analysed using the Seahorse extracellular flux analyser to measure their oxidative phosphorylation capabilities via the oxygen consumption rate (OCR) and their glycolytic capabilities via the extracellular acidification rate (ECAR). From this, the amount of ATP produced was calculated, and statistics performed using a two‐way ANOVA with a Sidak's post‐hoc test. **(B)** Radar plots illustrating the relative cytokine production of MNPs from blood or BAF in the absence of stimulation or when stimulated with LPS/R848 or POLY I:C. No statistical analysis was performed due to low replicates. **(C)** MNPs were analysed via flow cytometry for the expression of metabolic transporters (CD36, CD98), activation markers (CD163, CD206), and chemokine receptors (CCR2, CX3CR1) for control and COVID‐19 infected patients from blood (*n* = 9 and *n* = 7, respectively) and BAF (*n* = 10 and *n* = 7, respectively). Mann Whitney t tests were used for the statistics where *p* < 0.05 was deemed to be significant.

Given the established role of viral infection and an altered immunometabolic profile, we next considered whether metabolic dysregulation occurs upon SARS‐CoV‐2 infection. Interestingly, both peripheral and airway MNPs from severe COVID‐19 patients had compromised metabolism with decreased levels of both glycolysis and oxidative phosphorylation (Figure 2A). Due to restrictions in the BAF sample volume obtained, it was only possible to isolate MNPs from a single intubated patient for extracellular flux analysis. To establish whether the reduced metabolic profile impacted on effector function, the MNPs were left unstimulated or stimulated with LPS/R848 and poly I:C for 24 h (Figure 2B; Figure S3). Cytokine measurements from BAF‐derived MNPs were available from a single sample and are therefore presented as an exploratory observation without statistical inference but noting they show that BAF‐derived MNPs from SARS‐CoV‐2 infected patients had reduced cytokine production.

To determine why the MNP metabolic response was perturbed with SARS‐CoV‐2 infection, MNPs were analysed for the expression of various metabolic transporters and activation markers. Figure 2C quantifies CD36, CD98, CD163, CD206, CCR2 and CX3CR1, while additional markers (e.g., CD11b, CD209, CCR10, CXCR3, CD71, CD220) which were measured but not significant are presented in Figure S4. In the severe COVID‐19 cohort, peripheral blood monocytes had significantly lower expression of the metabolic transporters CD36 (fatty acids) and CD98 (long chain neutral amino acids) compared to control patients (Figure 2C; Figure S4). In addition, MNPs also had lower expression of the chemokine receptors CCR2 (blood only) and CX3CR1 (airways and blood), suggesting concomitant reductions in trafficking capabilities. While CD36 and CD98 expression were unchanged on airway MNPs, in addition to significantly reduced CX3CR1 in COVID‐19 patients these cells also expressed lower levels of the activation markers CD163 and CD206 in comparison to controls, suggesting a loss of M2‐like function. While there are limitations reflecting the number of samples that yielded enough isolated cells for the bioenergetics analysis assays and are therefore exploratory, overall, our data describe that MNPs from patients with SARS‐CoV‐2 infection are metabolically, phenotypically, and functionally dysregulated.

### 2.3 Differential Origins of BAF‐Derived EVs From SARS‐CoV‐2 Patients

EVs are becoming increasingly recognised as complex mediators of immunomodulatory effects on various cell types (Beez et al. 2019, Gärtner et al. 2018), capable of interacting with monocytes through surface receptors, such as CD36 (Cauvi et al. 2024), and inhibition of the CCL2/CCR2 (Liang, X. et al. 2025). Furthermore, EVs are a key component of biological fluids (Caby et al. 2005, Keller et al. 2011, Lässer et al. 2011, Pisitkun et al. 2004, Poliakov et al. 2009), capable of regulating processes at distant sites from the tissue of origin. As an example, EVs present in plasma of COVID‐19 patients have been shown to modulate lymphocyte responses (George et al. 2023). Due to the primary location of inflammation in COVID‐19 infection is the airways, we sought to determine whether airway‐derived EVs might have a role in the dysregulated phenotype evident in airways MNPs in severe COVID‐19 patients.

**FIGURE 3:**
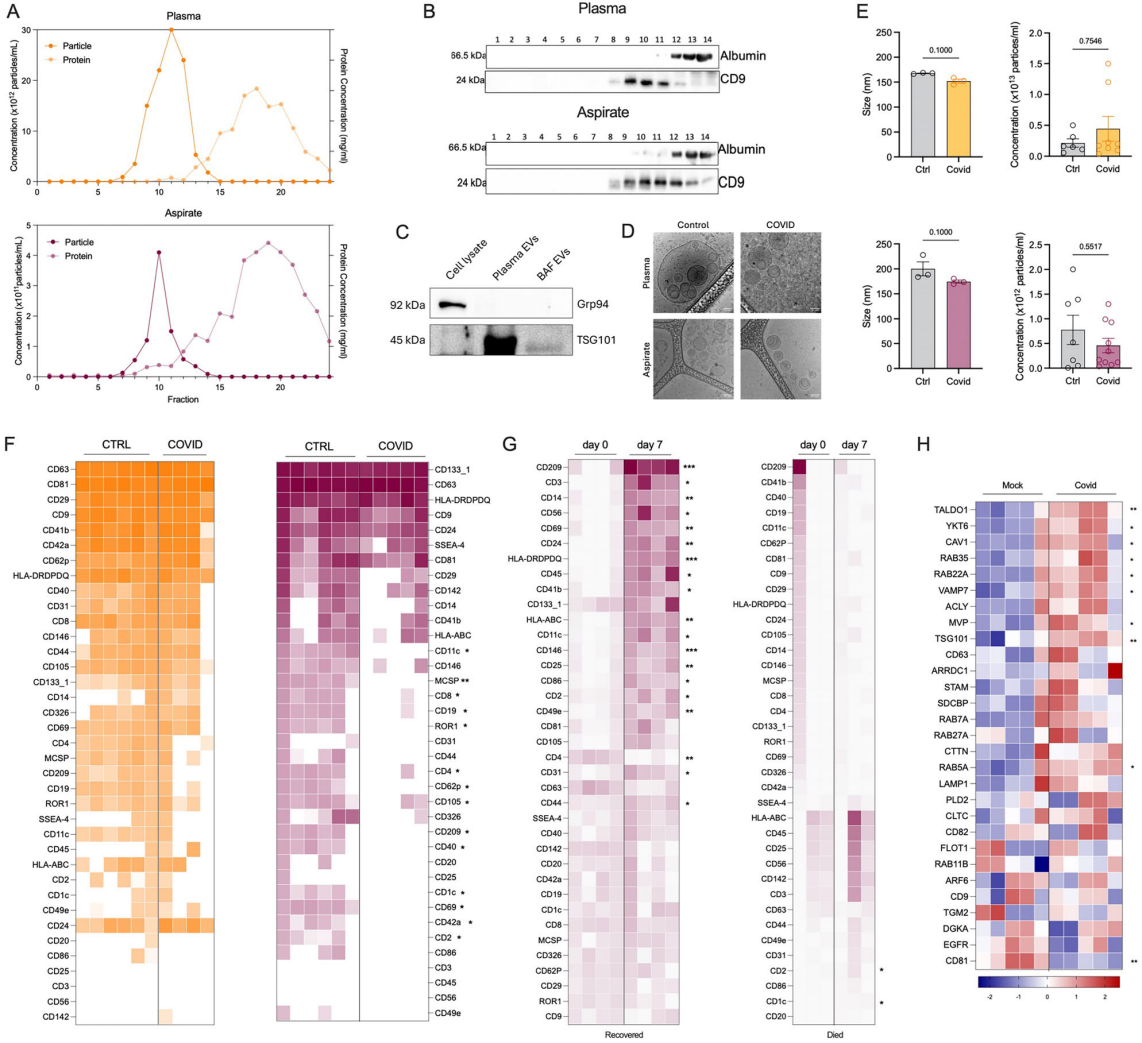
**Characterisation of sEVs. (A)** Characterisation of SEC fractions by particle and protein concentration from plasma and BAF. Particle concentration was analysed using the ZetaView, and protein concentration by DC assay. **(B)** Characterisation of SEC fractions by Western Blot. Equal volumes (15 µL) of each fraction (1–14) were loaded to determine their respective sEV content (CD9) and non‐sEV associated protein (Albumin). **(C)** Western blotting of concentrated sEVs was also used to confirm purity with a negative control (Grp94) and positive control (TSG101). **(D)** Cryo‐EM imaging shows sEVs with distinct bilayer membranes. **(E)** Concentration and mean size of sEVs from control and COVID‐infected patients. **(F)** MACSPlex was used to measure the surface markers present on sEVs, which provides information on their cellular provenance for control and COVID‐19 patients from whole blood (*n* = 6 and *n* = 4, respectively) and BAF (*n* = 6 and *n* = 5, respectively). **(G) s**EVs were analysed again using MACSPlex for their surface markers for comparison of matched day 0 and day 7 samples for patients with SARS‐CoV‐2 infection of those who recovered (*n* = 4) or died (*n* = 3). These patients were selected as they were discharged or died soon after day 7. **(H)** Markers of sEV biogenesis were extracted from deposited data by Mulay et al. (Aubertin et al. 2016) from transcriptomics of lung organoids which were mock‐ or COVID‐infected (*n* = 6/group). Red depicts higher expression, and blue lower. All statistics were performed using a Mann‐Whitney or paired t test where *p* <0.05 was deemed to be significant.

We first isolated EVs from plasma and BAF and characterised them in accordance with guidelines published within the Journal of EVs (Welsh et al. 2024). Individual fractions from the EV isolation were analysed for particle and protein concentration (Figure 3A) and tetraspanin (CD9) and albumin expression (Figure 3B). In addition, we further confirmed concentrated EV purity with a negative control (Grp94) and positive control (TSG101) (Figure 3C), noting that that there are fewer EVs in the BAF sample than the plasma, in line with the particle and protein concentration. Cryo‐EM images indicate that heterogenous EVs were isolated from all sample types with clear bi‐layer membranes visible, and no virus was detected in the images captured or the wider scan of the samples (Figure 3D). Size and overall concentration of pooled fractions (F8‐11) did not differ for EVs isolated from either plasma or BAF for control versus COVID‐19 (Figure 3E). While there are no clearly defined upper and lower size cut off limits (Welsh et al. 2024), for the purposes of this study given the EVs isolated are less than 200 nm by the characterisation methods used herein we will refer to them as small EVs (sEVs).

Having established that sEVs were present in the airways as well as the blood, the cellular provenance of sEVs at the two sites was then explored using the MACSPlex EV kit (Figure 3F; Figure S4–S6). For plasma‐derived sEVs there were no significant differences in any of the cell markers used to compare sEVs from control versus COVID‐19 patients (Figure 3F). In contrast, for BAF‐derived sEVs several key surface markers—platelet and endothelial markers (CD42a, CD62p, CD105), immune‐associated (CD1c, CD2, CD11c, CD69, CD209), and lymphocyte markers (CD4, CD8, CD19, CD40)—were decreased significantly in the BAF‐derived sEVs from COVID‐19 patients compared to control sEVs (Figure 3F), suggesting a possible change in overall proportion of sEVs from these cell types detectable within COVID‐19 patient plasma.

Some patients recovered from infection and were extubated and eventually discharged, while others did not recover, enabling us to determine, in a distinct analysis, if the altered phenotype shown in Figure 3F differed in patients who recovered versus those who died. In patients who recovered, expression of most cell provenance markers was restored on sEVs collected 7 days post‐intubation (Figure 3F; Figure S7). Platelet and endothelial (CD31, CD41, CD146), immune‐associated (CD2, CD11c, CD14, CD45, CD49e, CD56, CD69, CD86, CD209, MHC Class I, MHC Class II), and lymphocyte (CD3, CD4, CD24, CD25, CD44) markers all increased significantly, so that on day 7 BAF‐derived sEVs of recovering patients now resembled those from COVID‐19‐free participants. For those who died (Figure 3G; Figure S8), the loss of cell provenance markers persisted at day 7, with only CD2 and CD1c having significantly elevated expression compared to day 0. This suggests that perturbed EV phenotype plays a role in the disease course in COVID‐19.

Given that environmental stressors such as hypoxia, serum‐deprivation and alcohol have been associated with increased secretion and changes to protein and lipid cargos of sEVs alongside altered activity using *in vitro* models (Aubertin et al. 2016, Erwin et al. 2023, Haraszti et al. 2019, Mukherjee et al. 2020, Ren et al. 2019), we then investigated if sEV biogenesis within the airways is affected by SARS‐CoV‐2 infection. To achieve this, we reanalysed deposited data from Mulay et al., who modelled infection of primary human lung epithelium with SARS‐CoV‐2 versus a mock infection and produced RNA sequencing data (Mulay et al. 2021), in order to provide contextual support for epithelial modulation of EV‐biogenesis machinery during infection. After normalising the data based on the internal controls, we extracted the values for key markers of EV biogenesis (Figure 3H). Multiple EV machinery markers were upregulated (TALDO1, YKT6, CAV1, RAB35, RAB22A, VAMP7, MVP, TSG101, RAB5A) upon infection with SARS‐CoV‐2, with only one downregulated (CD81). Collectively, these data indicate that the cellular machinery enabling EV production within the lung epithelium becomes dysregulated with SARS‐CoV‐2 infection and could account for altered EV phenotype in the airways of patients with severe COVID‐19.

### 2.4 Plasma‐Derived EVs From SARS‐CoV‐2 Infection Influence MNP Function but Not Cellular Metabolism

**FIGURE 4:**
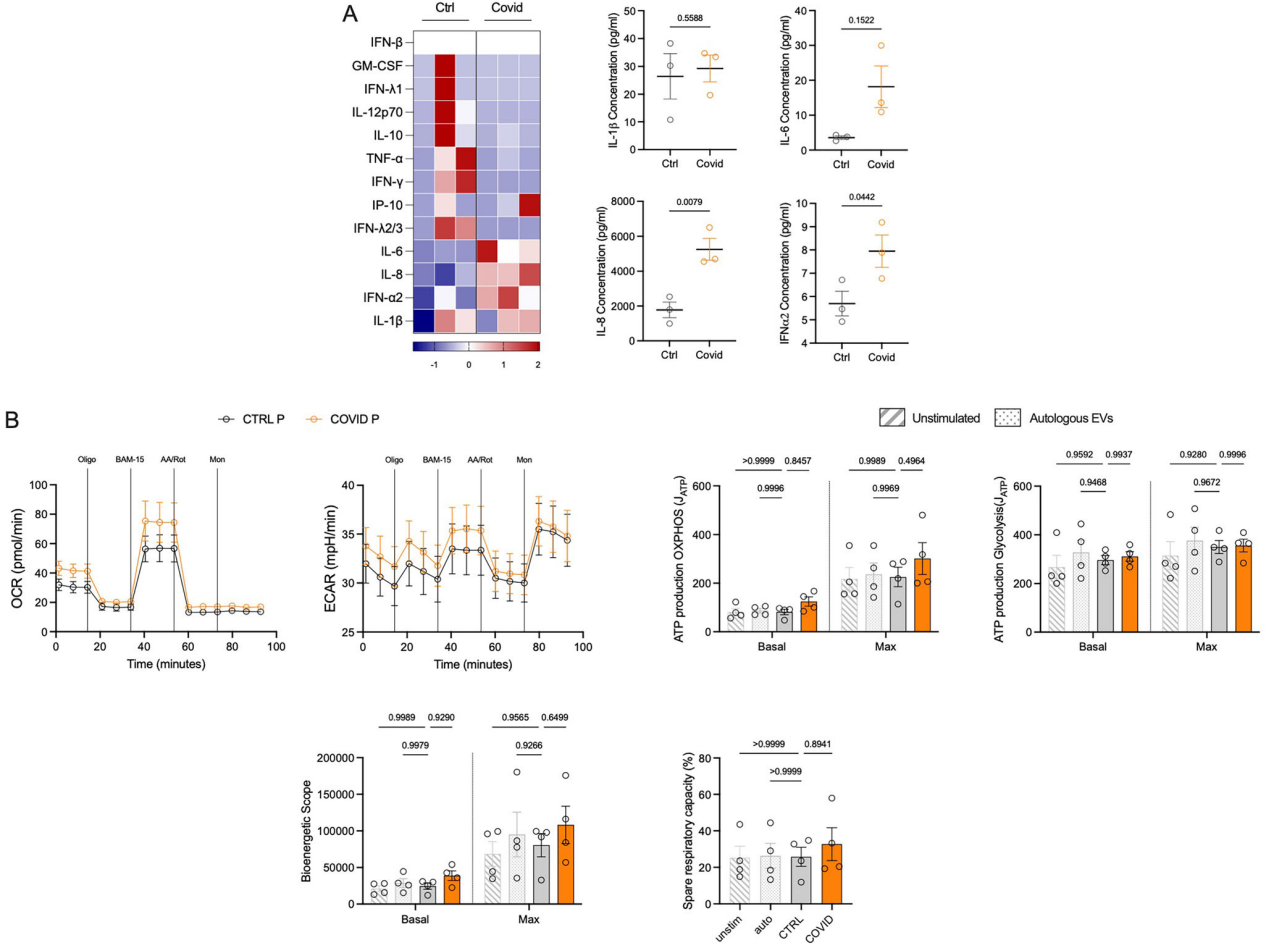
**sEVs from plasma were co‐cultured with monocytes from healthy volunteers**. Isolated monocytes from healthy volunteers aged 50+ years were co‐cultured with different patient sEVs at 1,000 sEVs/monocyte for 20 h. (A) Cytokines produced were measured using LEGENDPlexTM and flow cytometry for the effect of plasma‐derived sEVs. Heatmaps show if cytokines were secreted more (red) or less (blue), with those which were significant shown separately. (B) Metabolism of the monocytes was measured using OCR and ECAR after co‐culture with the plasma‐derived sEVs from control (grey) versus COVID‐infected (orange) patients. Bioenergetic measurements associated with the metabolism of the cells are shown with the unstimulated (no sEVs) and autologous sEVs of the monocyte donor, as extra controls. Measurements include ATP production by oxidative phosphorylation (OXPHOS) and glycolysis, bioenergetic scope, and spare respiratory capacity. Statistics were performed using either a two‐way or one‐way ANOVA with a Tukey's post‐hoc test and *p* <0.05 was deemed to be significant.

Given that altered sEVs from COVID‐19 patients could have different functional consequences, we next exposed monocytes isolated from healthy volunteers to sEVs derived from the plasma of ventilated patients with and without COVID‐19 to explore the functional effects of the sEVs on a relevant cell type (O'Neill and Pearce 2016, Sefik et al. 2022). Plasma‐derived sEVs from those with severe COVID‐19 versus those from control patients tended to have a pro‐inflammatory effect on monocytes with levels of IL‐8 and IFNα2 increased significantly (Figure 4A). This is in keeping with already described effects of circulating sEVs from severe COVID‐19 patients on increased effector cytokines levels from T cells (George et al. 2023).

As monocyte metabolism determines functionality (Jones et al. 2021, Rees et al. 2024) and the effect of circulating sEVs from severe COVID‐19 patients on T cells included increased glycolysis (George et al. 2023), we next determined if altered metabolism underpins this dysregulated cytokine response by monocytes. The effect of plasma‐derived sEVs from ventilated patients with and without COVID‐19 on glycolysis and oxidative phosphorylation of monocytes from healthy donors was therefore determined. Autologous sEVs were included as an additional control to ensure that sEVs did not interfere with the assay. Surprisingly, plasma sEVs did not have a significant effect on any of the glycolysis or oxidative phosphorylation parameters measured (Figure 4B.)

### 2.5 BAF‐derived EVs From SARS‐CoV‐2 Infection Alter the Proteome With Metabolic and Functional Consequences

**FIGURE 5:**
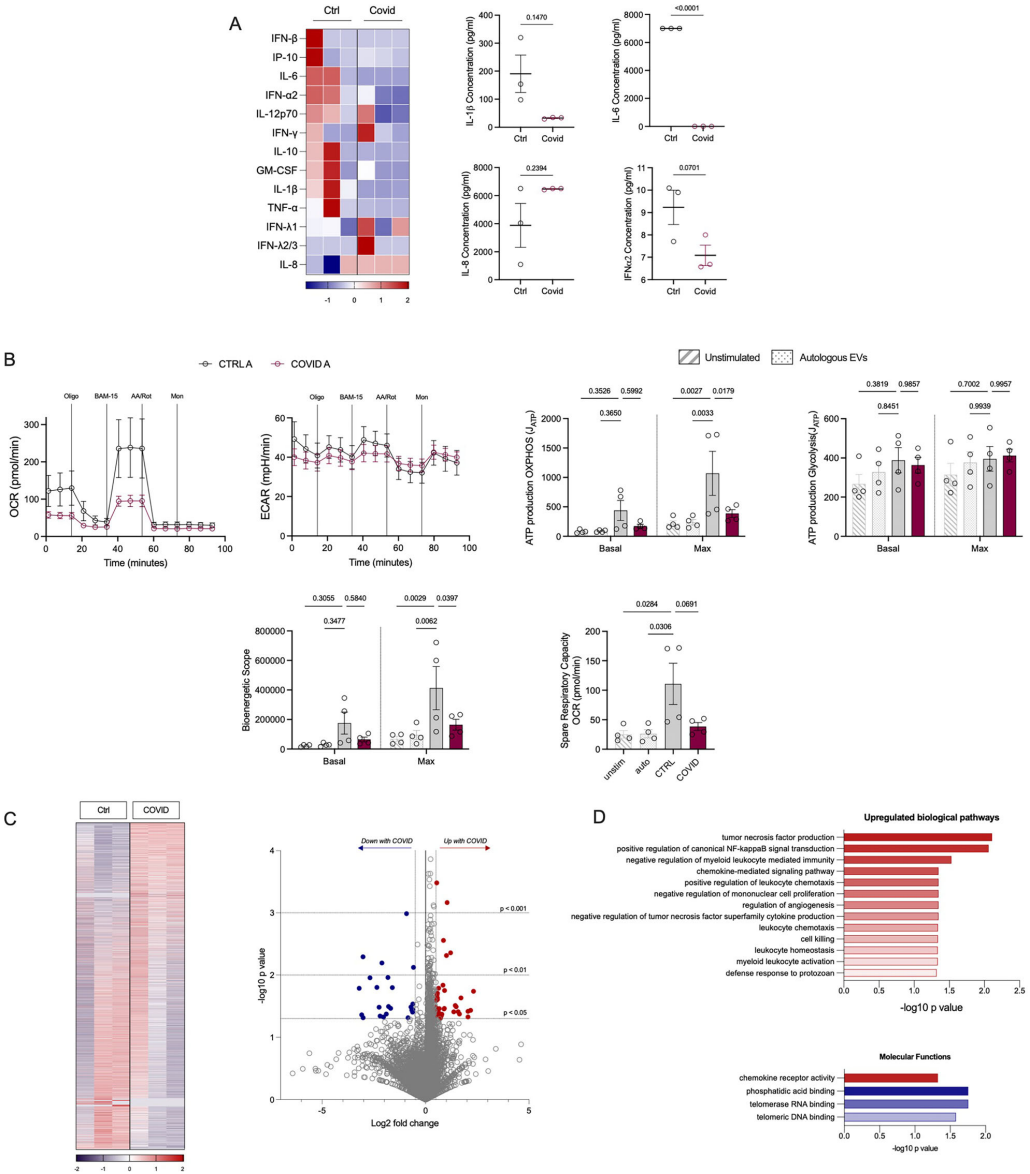
**sEVs from BAF were co‐cultured with monocytes from healthy volunteers**. Isolated monocytes from healthy volunteers aged 50+ years were co‐cultured with different patient sEVs at 1,000 sEVs/monocyte for 20 h. (A) Cytokines produced were measured using LEGENDPlexTM and flow cytometry for the effect of BAF‐derived sEVs. Heatmaps show if cytokines were secreted more (red) or less (blue), with those which were significant shown separately. (B) Metabolism of the monocytes was measured using OCR and ECAR after co‐culture with the BAF‐derived sEVs from control (grey) or COVID‐infected (maroon) patients. Bioenergetic measurements associated with the metabolism of the cells are also shown with the unstimulated (no sEVs) and autologous sEVs of the monocyte donor, as extra controls. Measurements include ATP production by oxidative phosphorylation (OXPHOS) and glycolysis, bioenergetic scope, and spare respiratory capacity. Statistics were performed using either a two‐way or one‐way ANOVA with a Tukey's post‐hoc test. (C) Cultured monocytes were analysed for proteomics with three replicates (same monocyte donor, three different sEV donors). Over 7000 proteins were identified and the copy number transformed to *z*‐scores for the heatmap. Proteins which were significantly different (*p* < 0.05, and FC ←0.5 or >0.5) between COVID‐19 and control sEV culture are highlighted as being up‐regulated in red or down‐regulated in blue on the volcano plot. (D) ORA analysis of the proteins which were significantly altered revealed biological pathways that were up‐regulated, and molecular functions that were either up‐regulated (red) or down‐regulated (blue); *p* <0.05 was deemed to be significant.

Thus far, we have demonstrated that BAF‐derived sEVs have an altered phenotype compared to plasma‐derived sEVs, so next we considered their impact on monocyte effector function and metabolism. While plasma‐derived sEVs from COVID‐19 patients had promoted a pro‐inflammatory response (Figure 4A) the converse was seen with BAF‐derived sEVs with an overall trend for decreased cytokine production in monocytes exposed to BAF‐sEVs from COVID‐19 patients although this was only significant for IL‐6 and IFNα2 (Figure 5A). This shows that the sEVs from the airways in SARS‐CoV‐2 infection dampen cytokine production which is a hallmark of MNP function in COVID‐19 and reminiscent of the airways MNP response to stimulation shown in Figure 2B. In addition, plasma sEVs from COVID‐19 patients did not significantly alter monocyte metabolism, whereas BAF sEVs from COVID‐19 patients impaired the ability of monocytes to upregulate oxidative phosphorylation, bioenergetic scope, and spare respiratory capacity compared to BAF sEVs from controls (Figure 5B).

To investigate the mechanisms by which the BAF‐derived sEVs modulate these effects on monocytes, we employed label‐free liquid chromatography mass spectrometry proteomics on whole cell lysates (Table S1). From this, approximately 7000 proteins were identified and of these, 61 were found to be significantly altered, which was defined as having a p value <0.05 and a log2 fold change of ←0.5 or >0.5 (Figure 5C; Table 1). Interestingly, 96 proteins were absent in at least 2 of the COVID‐19 samples compared to full expression by the control samples (Table S2), whereas 36 were absent in at least 2 of the controls versus the COVID samples (Table S3). ORA analysis revealed several biological pathways which were up‐regulated with severe COVID‐19, but none which were down‐regulated, in addition to altered molecular functions (Figure 5D). Interestingly, the up‐regulated pathways included many negative regulatory processes, such as myeloid leukocyte mediated immunity and TNF superfamily cytokine, in keeping with the dampened cytokine response and failure to upregulate cellular metabolism seen with the COVID‐19 BAF‐sEVs. The proteomic data reflects a complex reprogramming of monocytes exposed to BAF‐sEVs from COVID‐19 patients. These cells show features of suppressed inflammation, impaired energy metabolism, yet retain activity in chemotaxis and alternative immune pathways, reflecting the functional findings throughout this study. This could represent a dysfunctional state contributing to both immune suppression and chronic inflammation in COVID‐19.

**TABLE 1:** Significantly up‐ or down‐regulated proteins.

Down‐regulated protein	Log2 fold change	*p* value		Up‐regulated Protein	Log2 fold change	*p* value
ARHGAP10	−3.21	0.0163		WLS	2.32	0.0182
STBD1	−3.09	0.0435		CDC34	2.18	0.0368
ATP11C	−3.02	0.0051		TMEM41A	2.05	0.0382
NOD2	−3.02	0.0485		SDS	2.05	0.0470
USP33	−2.69	0.0111		PF4	1.72	0.0232
ATP13A2	−2.35	0.0158		ITM2B	1.64	0.0422
CCDC127	−2.25	0.0327		GNA12	1.58	0.0387
SLC36A4	−2.20	0.0453		GPNMB	1.48	0.0322
SMG6	−2.11	0.0064		TM6SF1	1.44	0.0308
HAUS6	−2.03	0.0471		LIPA	1.36	0.0389
APTX	−1.90	0.0422		EGLN1	1.21	0.0044
BTBD9	−1.82	0.0109		CX3CR1	1.04	0.0007
ZNF281	−1.79	0.0319		ARL14EP	1.01	0.0048
TMOD2	−1.69	0.0342		STUB1	0.92	0.0177
KCNK6	−1.61	0.0159		WDR82	0.91	0.0342
SPG7	−0.92	0.0010		PBX2	0.89	0.0349
ARID4B	−0.85	0.0483		CXCR2	0.86	0.0028
COG7	−0.71	0.0326		TSPAN32	0.84	0.0145
MED22	−0.66	0.0364		PTTG1IP	0.79	0.0426
SMG7	−0.63	0.0394		LGALS9	0.76	0.0453
GOLGA4	−0.62	0.0291		UTP6	0.72	0.0494
SESTD1	−0.61	0.0360		ING1	0.66	0.0346
MTHFSD	−0.58	0.0075		RRP8	0.63	0.0401
				SPRYD7	0.63	0.0163
				MITF	0.57	0.0196
				PXMP4	0.57	0.0247
				FMC1	0.57	0.0347
				EMC6	0.57	0.0387
				ANAPC7	0.57	0.0232
				GNPTG	0.55	0.0208
				SIAE	0.54	0.0442
				AGPAT5	0.54	0.0003
				TIMM22	0.54	0.0402
				DDX47	0.52	0.0406
				SCPEP1	0.52	0.0255
				TMEM9B	0.51	0.0333
				SLC38A7	0.50	0.0354
				LTF	0.50	0.0383

### 2.6 BAF‐EVs From Severe COVID‐19 Patients Are Taken up Less Preferentially and Carry a Significantly Different Cargo

A key role of EVs is to transport cargo between cells and tissues, modulating gene expression and initiating inflammatory processes (Smith et al. 2020, Xu et al. 2022), and so we investigated whether the interaction of sEVs with monocytes differs by blood or airway origin and if sEV cargo is altered with SARS‐CoV‐2 infection to explain the profound effects of BAF‐sEVs on MNP fate and function. The sEV fractions used throughout this study, including for lipidomics and miRNA analyses here, were taken from tetraspanin‐enriched, protein‐low SEC fractions. While plasma lipoprotein carryover cannot be fully excluded, airway aspirates contain comparatively low lipoprotein content and SEC effectively depleted abundant proteins (see fractionation profiles, Figure 3B).

**FIGURE 6:**
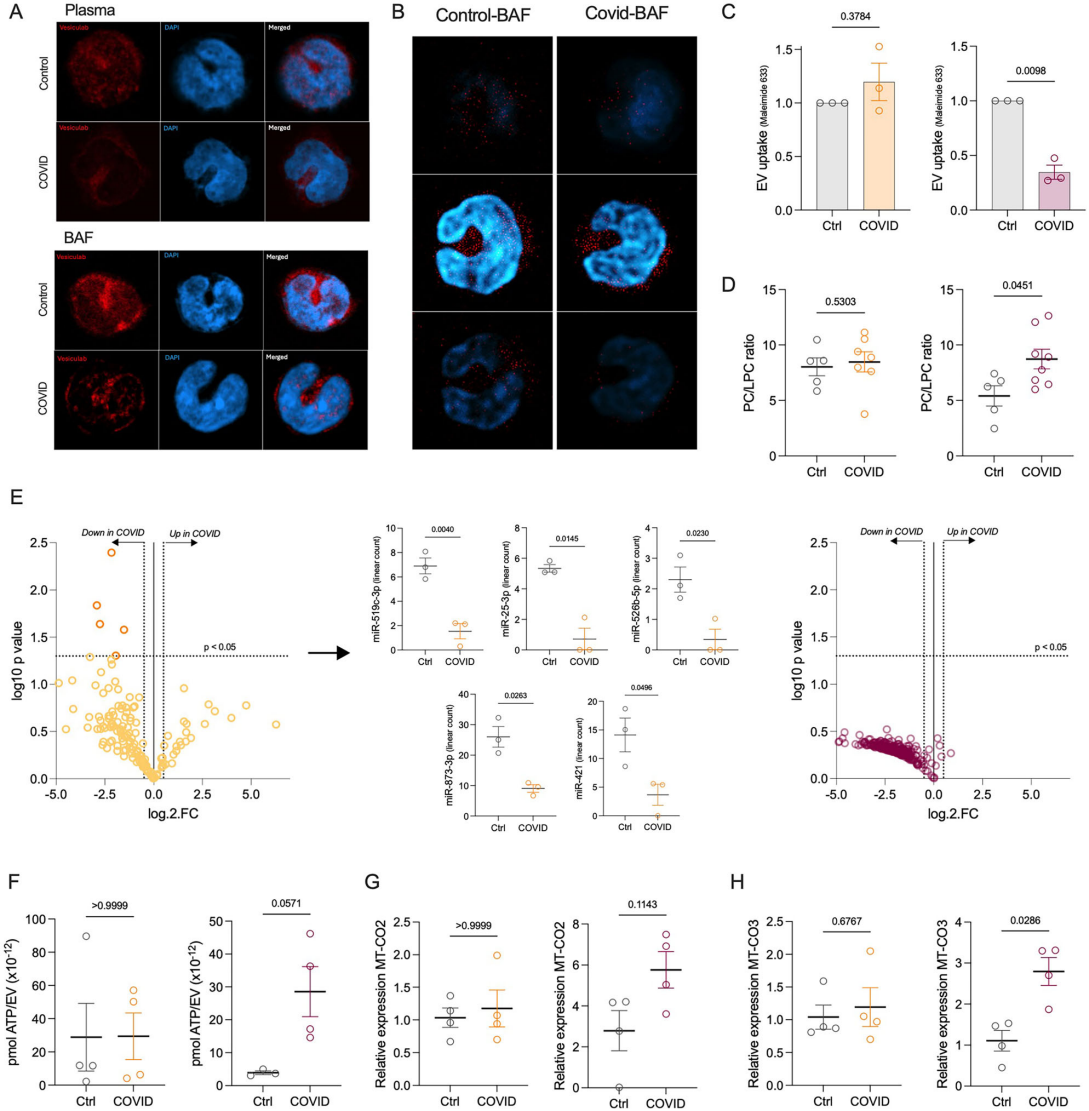
**Inspection of sEV cargo**. Plasma‐derived (orange) and BAF‐derived (maroon) intact sEVs were analysed. sEV uptake by monocytes was analysed using confocal microscopy with **(A)** Vesi‐Dye^TM^ LMB‐600 labelled sEVs (red) with cell nuclei stained with Hoechst 33342 (blue) and **(B)** Further inspection of BAF‐sEV uptake by monocytes using z‐stacks to show the localisation of the sEVs. **(C)** Maleimide Alexa Fluor 633‐labelled sEVs were used to quantify sEV uptake using flow cytometry (*n* = 3/group). **(D)** sEVs were investigated for their lipid composition using MALDI‐ToF to determine their PC/LPC ratio (*n* = 5 control plasma and aspirate, *n* = 7 COVID‐19 plasma, *n* = 8 COVID‐19 aspirate). **(E)** miRNA was measured in the sEVs using the NanoString^(R)^ nCounter^TM^ miRNA panel (*n* = 3/group) to illustrate miRNA species which were down‐ (log fold change < 0.5) or up‐ (log fold change > 0.5) regulated in sEVs from patients with COVID‐19. Those miRNAs which were significantly different (determined via Mann‐Whitney t test) are also depicted separately for the plasma‐derived sEVs. **(F)** ATP levels were measured and normalised per sEV present with the ATP colorimetric/fluorometric assay (*n* = 4). Mitochondrial DNA was measured using the relative expression of the **(G)** *MT‐CO2* (*n* = 4) and **(H)** *MT‐CO3* (*n* = 4) genes to NADH. To measure statistical significance, Mann–Whitney *t* tests were used and *p* <0.05 was deemed to be significant.

The interaction of Vesi‐Dye^TM^ LMB‐600 labelled sEVs with monocytes was monitored using confocal microscopy (Figure 6A,B; Figures S10–S13) including z‐stack to show intracellular localisation of the sEVs (Figure 6B shows BAF). While the interaction of sEVs with monocytes including the intracellular location of plasma‐derived sEVs did not differ between the groups with and without severe COVID‐19, for the BAF‐derived sEVs, both confocal microscopy and flow cytometry (Figure 6C; Figure S9 shows controls) revealed reduced interaction of the COVID‐19 BAF‐derived sEVs with the monocytes.

sEVs have a lipid bilayer, the composition of which influences membrane composition and integrity and allows sEVs to be taken up by target cells for cargo delivery. Phosphatidylcholine (PC) is the main component of lipid bilayers, and its derivative lysophosphatidylcholine (LPC) plays a multifaceted role in sEVs including causing positive curvature of the membrane (Abdolahi et al. 2022), and inducing inflammatory processes (Rees et al. 2023) such as macrophage migration, pro‐inflammatory cytokine production, and oxidative stress (Liu et al. 2020). The ratio between PC and LPC also provides a relatively simple measure of the contribution of lipids to the balance of pro‐ and anti‐inflammatory fluctuations, as we have shown previously (Rees et al. 2023). Mass spectrometry of sEVs from each group revealed that BAF‐derived sEVs from COVID‐19 patients have higher PC/LPC ratio than control patients, a difference not replicated in the circulating sEVs (Figure 6D). A higher PC/LPC ratio is indicative of less LPC suggesting they are less capable of being taken into the cell and therefore inducing an immune response (Kabarowski 2009, Kudo et al. 2022). Together, these findings support a role for altered COVID‐19 BAF sEV membrane composition in reduced EV uptake, providing a foundation for future experimental validation in expanded respiratory infection cohorts.

miRNAs are a key component of sEVs which regulate a cellular response, and we therefore employed a NanoString miRNA panel to profile >800 miRNA species in sEVs from all study groups (Figure 6E). A subset of plasma sEV miRNAs were down‐regulated in SARS‐CoV‐2 derived patients compared to controls; full lists are provided in Table 2. The list includes miR‐421 which has been found by others to be severely down‐regulated in the blood of SARS‐CoV‐2 patients at admission (Abdolahi et al. 2022). In airway sEVs, we observed a global miRNA reduction.

**TABLE 2:** Roles of the down‐regulated miRNA found in plasma‐derived sEVs from SARS‐CoV‐2 patients.

miRNA	Role
miR‐519c‐3p	promotes tumour growth (Wang et al. 2019)
miR‐25‐3p	promotes macrophage autophagy (Yuan et al. 2023), and endothelial permeability and angiogenesi (Zeng et al. 2018)
miR‐526b‐5p	targets c‐Myc and Foxp1; predominantly expressed in placental tissue, and is a tumour suppressor (Luo et al. 2023)
miR‐873‐3p	targets HDAC4 (Malavika et al. 2020)
miR‐421	down‐regulates ACE2 expression (Lambert et al. 2014), involved in hypoxia and lung injury (Islam and Khan 2020), and found to be severely down‐regulated in the blood of SARS‐CoV‐2 patients at admission (Abdolahi et al. 2022)

As a DAMP rich cargo has been suggested for sEVs in stressed microenvironments (Buzas 2023), we next measured levels of ATP and mtDNA in the sEVs from the different locations and study groups. BAF‐derived, but not plasma‐derived, sEVs from COVID‐19 patients had higher levels of intravesicular ATP (Figure 6F), and mitochondrial DNA, measured via expression of *MT‐CO2* and *MT‐CO3* genes (Figure 6G‐H). It is important to note that sEVs isolated via SEC here (∼150 nm), are too small to hold intact mitochondria (also confirmed in the cryo‐EM images in Figure 3C), so these must be true representatives of mtDNA. Together, these show that the sEVs from BAF of SARS‐CoV‐2 infected patients are intrinsically different from control patients with altered biogenesis (Figure 3) and altered cargo (Figure 6).

## 3 Discussion

This study provides significant insights into the pathogenesis of severe COVID‐19, specifically focusing on the metabolic and functional alterations in MNPs and the role of sEVs in mediating airways inflammation revealing a novel viral immunomodulatory strategy. Although ATP and mtDNA are classically pro‐inflammatory DAMPs, the composite features of COVID‐19 airway sEVs—higher PC/LPC ratio, global miRNA loss, and reduced uptake by target cells—together skew monocytes toward suppressed cytokine production and reduced OXPHOS, consistent with a multifactorial mechanism rather than canonical high‐dose DAMP activation.

Our initial characterisation of the participants blood corroborates previous reports that leukocyte populations are disrupted in the blood of COVID‐19 patients, with higher neutrophils and lower lymphocytes and monocytes (Qin et al. 2020). The elevated levels of IP‐10 in BAF samples from COVID‐19 patients indicate a localised hyperinflammatory response, which might exacerbate pulmonary damage. The depletion of other chemokines like MIG and I‐TAC could reflect a dysregulated immune response that fails to effectively control the infection (Blanco‐Melo et al. 2020). Of the soluble receptors typically associated with SARS‐CoV‐2 entry into cells, only soluble NRP‐1 was found to be decreased in the plasma of patients with COVID‐19. This is particularly interesting as a study involving the investigation of women with polycystic ovary syndrome (PCOS) found a negative correlation between plasma NRP‐1 and SARS‐CoV‐2 susceptibility (Moin et al. 2021).

Our study reveals that severe SARS‐CoV‐2 infection *in vivo* induces profound metabolic reprogramming in both peripheral blood and airway MNPs. This is consistent with the notion that immune cell metabolism is intricately linked to their function (Jones et al. 2021, Rees et al. 2024), and other studies have shown that MNP metabolism is impaired in severe (but not mild) SARS‐CoV‐2 infection (Shao et al. 2022), and that glycolytic activity is key for airway macrophage‐mediated lung homeostasis and regulation of inflammation in the airways (Albers et al. 2024). The decreased expression of metabolic transporters such as CD36 and CD98 in peripheral monocytes suggests a reduced capacity for using fuel sources other than glucose, which could impair their plasticity and adaptability in response to infection (Geeraerts et al. 2017) and the plasma metabolome has been shown to become restricted in the availability of nutrient metabolites in severe disease (Su et al. 2020). Additionally, the diminished expression of chemokine receptors CCR2 and CX3CR1 indicates compromised trafficking capabilities, potentially hindering effective immune surveillance and response, and an inability to replenish alveolar macrophages. In the BAF macrophages, lower expression of activation markers CD163 and CD206, along with reduced CX3CR1, points to an impaired activation state, which might limit their ability to mount an adequate immune response. Other studies have shown that in comparison to mild infection, severe COVID‐19 results in a suppressed myeloid compartment, postulated to be due to emergency myelopoesis (Schulte‐Schrepping et al. 2020, Silvin et al. 2020). This metabolic and functional impairment of MNPs likely contributes to the ineffective immune response observed in severe COVID‐19 cases, and we show here that this is most likely driven by sEVs within the local airways.

sEVs play a key role in maintaining pulmonary homeostasis through regulation of inflammatory responses and are crucial for host defence (Bourdonnay et al. 2015, Fujita et al. 2015). Here we have shown their importance within the COVID‐19 airways as key contributors to the dysregulated inflammation that occurs. Altered membrane composition in COVID‐19 airway sEVs is indicated by an absence of many cellular provenance markers and higher PC/LPC ratio that is implicated in a reduced capacity to induce immune responses (Donoso‐Quezada et al. 2021, Law et al. 2019). This is evidenced by the reduced ability of BAF‐sEVs from severe COVID‐19 patients to produce pro‐inflammatory mediators in our model, and the reduced chemokine receptor expression seen *ex vivo*. BAF‐sEVs in COVID‐19 also had a dramatically different cargo with a global reduction in miRNAs that are well characterised as a key cargo subtype of sEVs (De Jong et al. 2012, Groot and Lee 2020) and specifically of macrophage polarisation (Lee et al. 2019, Li et al. 2021). Such alteration in sEV phenotype and cargo could be explained by viral‐induced interference with the Endosomal Sorting Complex Required for Transport (ESCRT) pathway, and several other cargo sorting proteins, where cellular virus could hijack these pathways for their own replication and subsequent release from cells (Lin et al. 2022). This is evidenced in analysed deposited data where several key proteins involved in these pathways including TSG101^6^, are upregulated in a COVID‐19 mouse model. This would result in an increase in sEVs from virally infected cells, therefore altering the detectable proportion of sEVs from other cells (such as endothelial, immune‐associated and lymphocytes). Determination of sEV cell‐of‐origin, however, remains one of the most significant challenges with sEV research, when working with complex biofluids such as plasma.

Higher levels of DAMPs such as ATP and mitochondrial DNA which can further perpetuate inflammation and tissue damage (Abu et al. 2021, Wu et al. 2021) were seen in severe COVID‐19 BAF‐sEVs. The upshot of this altered cargo is an increase in various negative regulatory pathways within monocytes such as myeloid leukocyte mediate immunity and TNF superfamily cytokine production which account for the reduced cytokine production and altered metabolism we observed. This is possibly a mechanism to control hyperinflammation, coupled with the negative regulation of MNC proliferation for which oxidative phosphorylation would be crucial. Up‐regulation of NF‐κB signalling, leukocyte chemotaxis and chemokine‐mediated signalling, along with the observed increase in IL‐8 production, suggests that the monocytes are still involved in recruiting other leukocytes and activating inflammation through alternative pathways, which is corroborated with the elevated number of neutrophils observed *ex vivo*. While a limitation is that we have modelled this in monocytes of healthy donors, it is reminiscent of the response seen here in MNPs from the severe COVID‐19 airways. Future mechanistic perturbation studies, particularly those requiring larger sEV quantities or targeted pathway manipulation, will be pursued in a broader follow‐on‐on programme incorporating sEVs from other severe respiratory infections to determine whether similar immunometabolic reprogramming signatures extend beyond SARS‐CoV‐2. The phenotypic and cargo differences observed in BAF‐sEVs from severe COVID‐19 cases should be further investigated by examining sEVs directly from their cells of origin. This could clarify whether the differences arise from broad virus‐induced cellular changes or from specific disruptions in sEV biogenesis. Access to suitable tissue for subsequent cellular dissociation, especially considering health and safety requirements relating to SARS‐CoV‐2 infection, present a major challenge and are beyond the scope of this study.

The average age of the ventilated patients included in this study was 59. Our study has observed several hallmarks of ageing which suggest that infection with SARS‐CoV‐2 could accelerate cellular senescence—down‐regulation of telomerase activity (proteomics), mitochondrial dysfunction (oxidative phosphorylation decrease), altered intercellular communication (chemokine‐mediated communication), and deregulated nutrient‐sensing (*ex vivo* MNP differences in CD36 and CD98) (López‐Otín et al. 2023). Additionally, aged MNPs have been shown in mice to enhance angiogenesis with resulting increased susceptibility to injury‐associated angiogenesis in mice (Kelly et al. 2007, Liu et al. 2023). We show through the proteomic data that COVID‐19 BAF‐derived sEVs upregulate angiogenesis in monocytes. Other studies have shown a hypercoagulable state driven by neutrophils and monocytes in SARS‐CoV‐2 infection, leading to endothelial dysfunction and thromboinflammation, that play a critical role in progression of disease, particularly in those who die (Miggiolaro et al. 2023).

To ascertain how critical this sEV phenotype is to disease progression we took advantage of serial airways samples from severe COVID‐19 patients who recovered versus those who died. Recovery and eventual discharge were associated with recovery of the sEV phenotype whereas in patients who went onto die the sEV phenotype remained in disarray. The findings from this study therefore have important clinical implications. Understanding the metabolic and functional alterations in MNPs and the role of sEVs in severe COVID‐19 could inform the development of targeted therapies. Interventions aimed at modulating MNP metabolism or inhibiting the effects of sEVs could potentially ameliorate the hyperinflammatory state and improve patient outcomes.

In conclusion, our study highlights the critical role of sEV‐mediated communication and metabolic reprogramming especially in the airways during the pathogenesis of severe COVID‐19. By furthering our understanding of these processes, we can better target the underlying mechanisms driving hyperinflammation and develop more effective therapeutic strategies.

## 4 Subject Details

### 4.1 Sex as a Biological Variable

Both male and female human volunteers were involved in this study, with similar findings reported for both sexes.

### 4.2 Patient Samples

Matched arterial blood (collected into one 9 mL heparinised Vacuette ^TM^ [Greiner Bio‐one, Frickenhausen, Germany) and bronchial secretion samples were collected from patients with an arterial line and requiring intubation and mechanical ventilation. The first sample was collected on days 0–3 post‐ventilation. A bronchial secretion sample was also taken seven days after the first. COVID‐19 patients tested positive for SARS‐CoV‐2 at the time of sample collection, and control samples were SARS‐CoV‐2 negative. The presence of the virus was tested with a SARS‐CoV‐2 nasopharyngeal swab PCR. Participants lacked the capacity to provide informed consent, so a nominated consultee declared it was appropriate for the participant to be in the study. The nominated consultee was a consultant working on ICU with no involvement in the research study. Further declaration was sought from a personal consultee, such as a family member. Once participants regained capacity, they were invited to provide retrospective consent. Those who wished to withdraw had all data and samples collected destroyed. Ethical approval was obtained from a Health Research Authority Research Ethics Committee (20/NW/0452). The demographics of participants involved in this research are presented in Figure 1A.

### 4.3 Healthy Volunteers

Human peripheral blood (up to 120 mL) was collected from healthy volunteers into heparinised Vacuettes^TM^. All samples were collected with informed written consent and ethical approval obtained from Swansea University Medical School (SUMS) Research Ethics Committee (RESC), project reference 2022‐0029. The demographics of participants involved in this research are presented in Figure 1A.

## 5 Methods

### 5.1 Blood and Monocyte Isolation

Heparinised anti‐coagulated blood was centrifuged at 1800 *x g* for 10 min at room temperature; the plasma was removed and replaced with PBS. Plasma was further centrifuged at 3000 *x g* for 10 min to become platelet‐poor plasma and stored at ‐80°C for molecule analysis and sEV isolation.

MNCs were isolated by density gradient centrifugation using Lymphoprep^TM^ (Stem Cell Technologies, UK), and washed with RPMI 1640 and Glutamax (Life Technologies, Paisley, UK). Monocytes were isolated from MNCs with CD14 magnetic microbeads (Miltenyi) according to the manufacturer's instructions, using MACS LS manual columns (Miltenyi).

### 5.2 Bronchial Aspirates and Macrophage Isolation

77 mg/ml of dithiothreitol (Pierce^TM^ DTT No‐Weigh^TM^ Format [Thermo Scientific] in phosphate‐buffered saline [PBS; Life Technologies]) was added to the aspirate sample, which was then agitated at room temperature for 30 min until the sample dispersed. An equal volume of PBS was added to the aspirate before filtering through a pluriStrainer, 100 µm. Filtered aspirates were centrifuged at 515 *x g* for 10 min to produce a cell pellet. The supernatant was removed and stored at −80°C for molecule analysis and EV isolation. The cell pellet was re‐suspended in PBS, used for flow cytometry, and macrophages were isolated with CD14 magnetic microbeads (Miltenyi) according to the manufacturer's instructions, using MACS LS manual columns (Miltenyi).

### 5.3 Cell Culture of MNPs from Ventilated Patients

Monocytes and macrophages (1.0 × 10^5^ cells/200 µL) in RPMI 1640 and Glutamax, 10% fetal bovine serum (FBS; Hyclone, Cytvia) and 2‐mercaptoethanol (2‐ME; 100 µM) were left unstimulated or stimulated with lipopolysaccharide (LPS; 10 ng/ml; Invitrogen), LPS and resiquimod (R848; 400 ng/ml; Invitrogen) or polysinosinic: polycytidylic acid (POLY I:C; 25 µg/ml; Invitrogen) at 37°C in 5% CO_2_‐in‐air for 24 h. Supernatants were harvested for cytokine analysis and stored at −20°C.

### 5.4 Cytokine Analysis

ELISAs were carried out on plasma and aspirate supernatants as per the manufacturer's instructions: ACE2, CD26, C147, NRP‐1 (DuoSets; R&D Systems, Bio‐Techne). Further cytokine analysis on plasma, aspirate supernatants and cell supernatants was done via a multiplex approach using kits from BioLegend (LEGENDplex^TM^). The pre‐defined panels used were: 13‐plex human inflammation 1 panel, 12‐plex human proinflammatory chemokine panel 1, 13‐plex anti‐virus response panel, and a 5‐plex type 1/2/3 interferon anti‐virus response panel. These assays were performed according to the manufacturer's instructions.

### 5.5 Flow Cytometry

All flow cytometry data was acquired using the ACEA NovoCyte flow cytometer and analysed using FlowJo^TM^ (version 10.1; BD Biosciences), where compensation was applied to address any spectral overlap. Appropriate controls were used: unstained and single stains to correct for fluorescence spillover, and isotype controls (Table 3). Quality control (QC) particles (Agilent) were used daily to reduce inter‐session instrument variability.

**TABLE 3:** Antibody details.

Marker & Clone	Isotype	Fluorochrome	Catalogue number
CD3 (HIT3a)	mIgG2a	FITC	300306
CD4 (OKT4)	mIgG2b	FITC	317408
CD8 (HIT8a)	mIgG1	FITC	300906
CD19 (HIB19)	mIgG1	FITC	302206
CD20 (2H7)	mIgG2b	FITC	302304
CD34 (561)	mIgG2a	FITC	343604
CD56 (MEM‐188)	mIgG2a	FITC	304604
FceR1 (AER‐37)	mIgG2b	FITC	334608
CD45 (HI30)	mIgG1	BV785	304048
HLA‐DR (L243)	mIgG2a	PerCP/Cy5.5	307630
CD14 (M5E2)	mIg2a	BV510	301842
CD16 (B73.1)	mIgG1	BV711	360732
CD98 (REA387)	hIgG1	APC	130‐127‐296
CD36 (5‐271)	mIgG2a	BV421	336230
CD71 (CY1G4)	mIgG2a	BV650	334116
CD220 (B6.220)	mIgG2b	PE	352604
CD11b (ICRF44)	mIgG1	BV605	301332
CD163 (GHI/61)	mIgG1	PE	333606
CD206 (15‐2)	mIgG1	Alexa flour 647	321116
CD209 (9E9A8)	mIgG2a	BV421	330118
CCR2/CD192 (K036C2)	mIgG2a	APC	357208
CX3CR1 (2A9‐1)	rIgG2b	BV421	341620
CXCR3/CD183 (G025H7)	mIgG1	BV605	353728
CCR10 (6588‐5)	hamster IgG	PE	341504
isotypes	hamster IgG	PE	400908
	mIgG1	BV510	400172
	rIgG2b	APC	400612
	mIgG1	BV605	400162
	mIgG1	PE	400112
	mIgG1	Alexa flour 647	400130
	mIgG2a	BV421	400260
	mIgG1	BV421	400158
	mIgG2a	APC	400222
	rIgG2b	BV421	400655
	mIgG2a	BV650	400266
	mIgG2b	PE	400314

Whole blood and aspirate cell populations were analysed using an 8‐colour immunophenotyping kit, human (Miltenyi Biotec, UK). This cocktail contains: anti‐CD3 PE (IgG1, clone REA613), anti‐CD4 VioBright^TM^ 667 (IgG1, clone REA623), anti‐CD8 APC‐Vio 770 (IgG1, clone REA734), anti‐CD14 VioBlue (IgG1, clone REA599), anti‐CD16 VioBright 515 (IgG1, clone REA423), anti‐CD19 PE‐Vio 770 (IgG1, clone REA675), anti‐CD45 VioGreen^TM^ (IgG1, clone REA747), anti‐CD56 VioBright 515 (IgG1, clone REA196). 7‐AAD staining solution was used to omit dead and apoptotic cells, and lysis of the red blood cells (RBCs) was carried out with the RBC lysis solution provided in the kit.

Blood MNCs and aspirate cells were treated with Human TruStain FcX^TM^ Fc receptor blocking solution (BioLegend). All antibodies used were obtained from BioLegend, except for CD98, which was from Miltenyi. Antibody information is detailed in Table 3.

### 5.6 Bioenergetic Analysis

Bioenergetic analysis was carried out using the Seahorse Extracellular Flux Analyzer XFe96 (Agilent Technologies). Monocytes and macrophages (2.0 × 10^5^ cells/well) in XF assay media (minimal Dulbecco's modified eagle medium [DMEM]; Agilent) supplemented with 5.5 mM glucose (Agilent), 1 mM pyruvate (Agilent) and 2 mM glutamine (Sigma) were seeded onto a Seahorse XFe96 Pro PDL‐coated culture microplate (Agilent) (from patient samples) or a Cell‐Tak^TM^ (22.4 µg/ml; Corning) coated microplate (Jones et al. 2015) (from healthy volunteers). Parameters for oxidative phosphorylation (OXPHOS) and glycolysis were measured simultaneously via oxygen consumption rate (OCR; pmoles/min) and extracellular acidification rate (ECAR; mpH/min) respectively with use of injections: oligomycin (1 µM), FCCP (1 µM), antimycin A and rotenone (both 1 µM) and monensin (20 µM) (all from Merck).

### 5.7 sEV Isolation

sEVs were isolated from platelet‐free plasma and BAF using size exclusion chromatography (SEC; Exo‐spin; Cell Guidance Systems, UK), according to the manufacturer's instructions. In brief, plasma and BAF were pre‐cleared of remaining cells and debris by centrifugation at 6000 *x g* for 10 min at 4°C. Exo‐spin midi columns were equilibrated with 20 mL of PBS and allowed to drain under gravity. Following equilibration, 1 mL of sample was added to the column, and 500 µL fractions were obtained following sequential additions of 500 µL of PBS. A total of 24 fractions were obtained from each sample. All isolations were normalised to 1 mL starting volume.

### 5.8 sEV Characterisation

Following isolation, the protein concentration of each fraction was assessed using DC protein assay (Bio‐Rad, USA), according to the manufacturer's instructions.

The particle size and concentration of each fraction were determined by nanoparticle tracking analysis (NTA) using the ZetaView (Particle Metrix, Germany). Briefly, the ZetaView was calibrated using an alignment solution containing 100 nm polystyrene beads prior to data acquisition. Fractions and sEV samples were diluted accordingly in PBS so the particle concentration was within the linear range of the instrument to avoid oversaturation of the camera. Measurements were taken using a 488 nm 40 mW embedded laser and analysed using the integrated ZetaView software. Three measurement cycles were performed in all 11 positions of the measurement cell with the following camera settings; shutter: 80, sensitivity: 100 and temperature: 25°C.

Immunoblotting was also used to determine the sEV content of each fraction by measuring CD9 and serum albumin, respectively. Briefly, fractions were diluted 1 in 4, and equal volumes (20 µL) were mixed with Laemmli buffer containing β‐mercaptoethanol. Samples were heated at 70°C for 10 min and cooled on ice. Proteins were separated using SDS‐PAGE on in‐house made 12% gels. Following SDS‐PAGE, semi‐dry transfer of proteins from gel to PVDF membrane was performed using the Trans‐Blot Turbo system (Bio‐Rad). Membranes were blocked for 1 h at room temperature with 0.05% Tween‐20 and 5% BSA in TBS. Subsequently, the membrane was incubated overnight at 4°C with primary antibodies; CD9 (13174S; Cell signalling, UK), Serum Albumin (MAB1455; R&D systems, USA), Grp94 (SC‐393402; Santa Cruz Biotechnology, USA), and TSG101 (SC‐7964; Santa Cruz Biotechnology, USA). Membranes were washed with 0.05% tween in TBS and further incubated for one hour at room temperature with an Anti‐Mouse (HRP‐linked) secondary antibody (7076S, Cell signalling). The membranes were further washed as previously in preparation for imaging. Amersham ECL Select Western Blotting Detection Reagent (GE Healthcare, USA) was applied to the membrane for imaging. The chemiluminescent signal was captured using ChemiDoc XRS+ (Bio‐Rad) and Image Lab software.

Following characterisation, the sEV‐containing fractions (F8‐11) were pooled and concentrated using Amicon Ultra‐2 50 kDa centrifugal filters (Merck, UK). Each sEV preparation was concentrated from 2 mL to 200 µL and subsequently analysed using NTA before storing at −80°C prior to downstream analysis.

### 5.9 sEV Origin and Surface Phenotype

A bead‐based multiplex flow cytometry assay was used to phenotype the surface of sEVs in different sample types (MACSPlex Exosome Kit; Miltenyi Biotech, Germany). Concentrated sEV samples (20 µg) were diluted with MACSPlex buffer to a total volume of 120 µL, and 15 µL of MACSPlex sEV capture beads (containing the different capture bead subsets) were added to each sample and incubated overnight on an orbital shaker (450 rpm) in the dark. Subsequently, samples were washed once by adding MACSplex buffer and centrifuged at 3000 *x g* for 5 min to remove residual unbound antibodies. 15 µL of sEV detection cocktail (5 µL CD9, 5 µL CD63, 5 µL CD81) was added to each sample and incubated for one hour on an orbital shaker (450 rpm) in the dark. Following two consecutive washes (as previously), samples were transferred to a 96‐well round bottom plate for acquisition on the ACEA NovoCyte flow cytometer. Data were acquired using the NovoExpress software and later analysed using FlowJo. Raw MFI values were background corrected using a PBS blank sample and normalised to the mean expression of tetraspanin sEV markers (CD9‐63‐81 total MFI).

### 5.10 Cryo‐EM

To prepare samples for cryo‐EM lacey carbon grids (Cu, 300 mesh) were glow‐discharged (45s at MED RF power level) in Harrick plasma cleaner. 4 µL of sample was applied to the carbon side of the EM grid, which was then blotted for 2.5 s at 95% humidity and plunge‐frozen into Leica GP2 plunger. Grids were imaged on a Jeol JEM‐2100 plus (Queen Mary University of London), at accelerating voltage of 200 kV equipped with Gatan OneView 16MP camera. Images were obtained using SerialEM software at low dose mode at 50k magnification, corresponding to 2.166Å/pixel with the defocus range set from ‐2.5 to ‐5 µm.

### 5.11 RNA Isolation

Total RNA was isolated from sEVs using the Norgen plasma/serum RNA purification Mini Kit using the manufacturer's instructions with slight modifications. Briefly, 200 µL of isolated sEVs were combined with 600 µL Lysis Buffer and 800 µL 100% ethanol to prepare an sEV lysate. A micro spin column was assembled, and the lysate was passed through the column by sequential centrifugations at 200 *x g*, 3500 *x g* and 14,000 *x g*, each for 1 min. The column was washed with 400 µL Wash Solution A and centrifuged for 14,000 *x g*. Washing was repeated three times with a final 2 min centrifugation at 14,000 *x g* to completely dry the column. RNA was eluted from the column by adding 10 µL Elution Solution A, let stand for 2 min and centrifuged for 2 min at 200 *x g* and 5800 *x g*. Isolated RNA was stored at ‐80°C until downstream analysis.

### 5.12 NanoString

A standardised volume of RNA was used to ligate the miRNA, which was subsequently used for the hybridisation reaction as per the manufacturer's instructions (NanoString), using the nCounter miRNA Expression Assay Kit. Hybridised samples were run on the nCounter SPRINT (NanoString, USA). Data was exported to nSolver (NanoString) where the miRNA was normalised based on the internal housekeeping probes. To determine the threshold of accurate detection, the average from the negative controls was calculated for each sample and removed from the targets. Only those with a minimum of 4 counts across the 6 samples (*n* = 3 per group) were included in the analysis. A *t*‐test on Excel was used, and significance was determined to be a p‐value less than 0.05 and a log 2‐fold change of ≥0.5 or ≤−0.5. The study was exploratory and based on a small cohort, with high‐dimensional data. Formal multiple‐comparison correction was not applied because of the small sample size, the high correlation between features, and the risk of eliminating biologically meaningful signals. Instead, raw p‐values were reported alongside an effect‐size threshold of log_2_ fold‐change ≥0.5 or ≤−0.5 to minimise trivial differences.

### 5.13 PC/LPC Determination

sEVs were used in duplicate for analysis with a matrix‐assisted laser deionisation (MALDI) time of flight (ToF) mass spectrometer as described previously (Rees et al. 2023). Briefly, 1 µL of sEVs were spotted onto a MALDI target plate (MTP target frame III, Bruker) and allowed to dry before the addition of 0.5 µL 9‐aminoacrinidine (9‐AA) matrix solution (10 mg/ml produced with a 1:1 w/v with 2‐propanol/acetonitrile [60/40, v/v]). Samples were analysed directly using an ultrafleXtreme MALDI TOF mass spectrometer (Bruker Daltonics) to acquire the spectra in the positive polarity. Five thousand single laser shots were summed to obtain each mass spectrum in the m/z range of 400–1000. Data was imported into R (Version 2023.12.1, RStudio) for pre‐processing using the MALDIquant package (Version 1.22.2) (Lee et al. 2019). Peaks were identified as specific lipids using their m/z value for which peak intensity was used for calculation of the ratio of the PC and LPC species, calculated as PC/LPC.

### 5.14 ATP Detection Assay

A set volume of sEVs (25 µL) was used with the ATP colorimetric/fluorometric assay kit (Merck) (Abu et al. 2021, Wu et al. 2021). The assay was performed as per the manufacturer's instructions for the more sensitive fluorometric reaction. The amount of ATP was calculated as pmol/µL from the standard curve and normalised to the number of sEVs present in each sample (calculated earlier with the ZetaView) to provide the pmol of ATP per sEV.

### 5.15 mtDNA PCR Assay

20 µL qPCR reactions were prepared using 10 µL Fast SYBR^TM^ Green Master Mix (ThermoFisher Scientific), 8 µL nuclease free water (NFW), 1 µL primer and 1 µL sEVs. qPCR was carried out using CFX Connect Real‐Time PCR Detection System (Biorad). The thermal cycling protocol was denaturation for 3 min at 95°C, followed by 39 cycles of 10 s at 95°C and 30 s at 60°C. List of primer sequences shown in Table 4. Analysis was carried out using the 2^−ΔΔCt^ method.

**TABLE 4:** **Primer sequences used for RT‐qPCR**. Cytochrome C oxidase subunit II (*MT‐CO2*); Human mt‐cytochrome C oxidase subunit III (*MT‐CO3*); nicotinamide adenine dinucleotide (NADH).

Gene	Forward primer 5′‐3′	Reverse primer 5′‐3′	Reference
***MT‐CO2***	ATGACCCACCAATCACATGC	ATCACATGGCTAGGCCGGAG	Pollara et al. 2018 (López‐Otín et al. 2023)
***MT‐CO3***	ATGACCCACCAATCACATGC	ATCACATGGCTAGGCCGGAG	Scozzi et al. 2019 (Liu et al. 2023)
**NADH dehydrogenase**	ATACCCATGGCCAACCTCCT	GGGCCTTTGCGTAGTTGTAT	Konaka et al. 2023 (Kelly et al. 2007)

### 5.16 EV Uptake by Monocytes

50 µL of isolated sEVs were stained for 1 h with 1 µL Vesi‐Dye LMB‐600 (Vesiculab, Nottingham UK) or with 0.15 µL C5‐maleimide‐Alexa 633 (ThermoFisher). Excess dye was removed using the Vesi‐SEC micro spin columns (Vesiculab, Nottingham UK), with samples centrifuged 1000 *x g* for 1 min.

Monocytes from healthy volunteers were cultured at 2.5 × 10^5^ cells/ 250 µL exosome‐free media (RPMI 1640 and Glutamax, 10% exosome‐depleted FBS [Gibco, Fisher Scientific] and 2‐ME) and were left unstimulated or stimulated with the stained sEVs (1 × 10^3^ / monocyte as is physiological in the circulation (Auber and Svenningsen 2022)) for 1 h either at 37°C in 5% CO_2_‐in‐air, or on ice to prevent uptake of EVs. Those with the Vesi‐Dye LMB‐600 dye were cultured directly onto chamber slides (Millicell EZ slide), whereas those with the Maleimide Alexa Fluor 633 were cultured in culture tubes. Cells with the Maleimide Alexa Fluor 633‐stained sEVs were washed with FACS buffer, centrifuged at 515 *x g* for 7 min, re‐suspended in 200 µL FACS buffer and analysed immediately on the flow cytometer as before. SARS‐CoV‐2 samples were normalised to the control sample per healthy donor.

Cells with the Vesi‐Dye LMB‐600 dye stained sEVs were fixed with 100% methanol and stained with Hoechst 33342 (ThermoFisher Scientific; Massachusetts, USA) for 15 min. Cells were washed 3 times with PBS, before all buffer was aspirated. The wells were removed, and coverslip mounted with Vectashield Antifade mounting medium (Vector Laboratories). Images were acquired using a Zeiss LSM 980 Airyscan microscope using the 63x objective. Images were processed using ImageJ (FIJI).

### 5.17 sEVs and Monocytes Co‐Culture

1.5 × 10^6^ monocytes from healthy volunteers were cultured in 1.5 mL of exosome‐free media (as detailed above) and left unstimulated or stimulated with sEVs (1 × 10^3^ / monocyte as previously) at 37°C in 5% CO_2_‐in‐air for 20 h. sEVs used include autologous plasma sEVs and the aspirate and plasma sEVs from the patient cohorts. Supernatants were harvested for cytokine analysis and stored at −20°C, and cells were used for bioenergetic or proteomic analysis.

### 5.18 Proteomics

*Sample preparation*: 7 × 10^5^ of monocytes cultured as above were washed with 1 mL HBSS and centrifuged at 17,000 x g at 4°C for 10 s twice. The pellet was snap frozen in liquid nitrogen for 10 s before storage at −80°C.

*S‐Trap sample processing*: Sample volume was increased to 100 µL by the addition of 1x S‐Trap lysis buffer (5% sodium dodecyl sulfate (Sigma‐Aldrich, #L4509‐10G), 100 mM tetraethylammonium bromide (TEAB, Sigma‐Aldrich, #T7408‐100ML)). The samples were then sonicated in a waterbath sonicator (frequency 37, power 80) for 5 min at room temperature. Protein quantification determined by Micro BCA (ThermoFisher Scientific, #23235). From each sample 100 µg of protein was processed using S‐Trap mini columns (Protifi, #CO2‐mini‐80). The default Protifi S‐Trap mini protocol was followed, modified such that the columns were washed 5 times with 100 mM TEAB in 90% methanol, and the samples were digested overnight with 2.5 µg of trypsin (1:40, Thermo Fisher, Pierce Trypsin Protease MS‐Grade, #90057) with a second digest with the same amount of trypsin for 6 h the following day. Peptides were extracted as detailed in the Protifi protocol and then dried under vacuum. The peptides were resuspended to 50 µL with 1% formic acid (Fisher Chemical, A117‐50). An aliquot of the peptides was then diluted 1:5 with 1% formic and then quantified by analysis on a Thermo QExactive.

*Data independent analysis (DIA) mass spectrometry*: Peptides were run on an Orbitrap Astral mass spectrometer (Thermo Scientific) coupled to a Vanquish Neo UHPLC system (Thermo Scientific) with LC buffers compromising of buffer A (0.1% formic acid) and buffer B (80% acetonitrile (VWR, 83640.290), 0.1% formic acid). The buffers were used to create a gradient (4‐35% B) for a 60SPD (samples per day) run where the peptides were eluted from a PepMap RSLC C18 column (Thermo Scientific PNES906) and RAW data was acquired in Data Independent Acquisition (DIA) mode. A scan cycle compromised a full MS scan with an m/z range of 380–980, resolution of 240,000, custom Automatic Gain Control (AGC) target of 500% and a maximum injection time (IT) of 5 ms. MS scans were followed by MS/MS DIA scans of dynamic window widths with an overlap of 0m/z. DIA spectra were recorded with a scan range of 150–2000m/z, custom AGC target of 500% and a maximum IT of 3 ms. Normalised collision energy was set to 25% with a default charge state set at 2. Data for MS scans were acquired in profile mode with MS/MS DIA scan events being acquired in centroid mode.

*Data analysis*: Analysis of the DIA data was carried out using Spectronaut (version 16.2.220903.53000, Biognosys, AG). The direct DIA workflow, using the default settings (BGS Factory Settings) with the following modifications was used: decoy generation set to mutated; Protein LFQ Method was set to QUANT 2.0 (SN Standard) and Precursor Filtering set to Identified (Qvalue); Cross‐Run Normalization was unchecked; Precursor Qvalue Cutoff and Protein Qvalue Cutoff (Experimental) set to 0.01; Precursor PEP Cutoff set to 0.1 and Protein Qvalue Cutoff (Run) set to 0.05.

For the Pulsar search the settings were: maximum of 2 missed trypsin cleavages; PSM, Protein and Peptide FDR levels set to 0.01; scanning range set to 300–1800 m/z and Relative Intensity (Minimum) set to 5%; cysteine carbamidomethylation set as fixed modification and acetyl (N‐term), deamidation (asparagine, glutamine), dioxidation (methionine, tryptophan), glutamine to pyro‐Glu and oxidation of methionine set as variable modifications. The database used was *H. sapiens* downloaded from uniprot.org on 2021‐10‐11 (77,027 entries).

Further data analysis was performed using Perseus (version 1.6.15.0, https://maxquant.net/perseus/) to generate copy numbers and in Microsoft Excel Office 365 to generate protein fold‐change values. Copy numbers were exported onto R (Version 2024.09.1 + 394, RStudio) to create *z*‐score values and for further analysis. Significant protein changes were determined to be *p* ≤ 0.05 by *t* test and FDR correction, and with a fold change of ≤ −0.5 or ≥ 0.5. Over‐representation analysis (ORA) was used to determine which pathways are altered, with the significant protein changes identified with the clusterProfiler package (version 4.12.6) (Wu et al. 2021, Yu et al. 2012) on R used with the enrichGO function and the database for *H. sapiens* (org.Hs.eg.db downloaded from Bioconductor.org).

### 5.19 Statistics

Statistical analysis was performed using GraphPad Prism V9 (Dotmatics). Data are represented as the mean ± standard error of the mean (SEM). To test for normality, the one‐sample Kolmogorov‐Smirnov (K‐S) test. A Mann–Whitney test was used if the data was non‐parametric for comparison between two sets of data, or a two‐way ANOVA for further groups with a Šidák's multiple comparisons post‐hoc test. A *p* value of 0.05 was determined to be significant. Specific details and replicate numbers can be found in the figure legends. Due to the low volume of sample from most patients, some experiments have less than an *n* = 3.

## Author Contributions

**April Rees**: conceptualization, investigation, writing – original draft, methodology, validation, visualization, writing – review and editing, formal analysis, project administration, data curation. **Oliver Richards**: investigation, conceptualization, writing – original draft, methodology, validation, visualization, writing – review and editing, formal analysis, data curation. **Molly E Raikes**: investigation, formal analysis, validation, writing – review and editing. **Megan Chambers**: investigation, writing – review and editing, formal analysis. **Sophie G Reed**: Investigation, formal analysis, validation, writing – review & editing. **Ceri Battle**: investigation, data curation, resources, writing – review and editing, project administration, conceptualization, funding acquisition. **Hannah Toghill**: investigation, writing – review and editing, project administration, resources. **Luke Newey**: conceptualization, investigation, project administration, resources, writing – review and editing, funding acquisition. **Tyler J Joseph**: investigation, formal analysis, writing – review & editing. **Haiyan An**: formal analysis, writing – review and editing, methodology. **Hui Zhang**: methodology, visualization, writing – review and editing. **Iain Perry**: methodology, validation, writing – review and editing, formal analysis. **Jason Webber**: methodology, validation, writing – review and editing, supervision, resources. **Nicholas Jones**: conceptualization, investigation, funding acquisition, writing – review and editing, resources, supervision, methodology. **Catherine A Thornton**: conceptualization, funding acquisition, project administration, resources, software, supervision, writing – original draft, review & editing.

## Funding

This work was funded by the Medical Research Council (MRC), Tackling COVID‐19 project under grant MR/V037013/1.

## Conflicts of Interest

The authors declare no conflicts of interest.

## Supporting information



Supporting material: jev270288‐SUP‐0001‐Figures.pdf

Supporting material: jev270288‐sup‐0002‐Tables.xlsx

## Data Availability

NanoString data have been deposited at GEO, and proteomics data at PRIDE, and are publicly available as of the date of publication. Accession numbers are listed in the key resources table. This paper does not report original code. Any additional information required to reanalyse the data reported in this paper is available from the lead contact upon request.
