# Supplementary material for: DAMP Laden Extracellular Vesicles From the Airways of Patients With Severe SARS‐CoV‐2 Respiratory Infection Compromise Inflammation and Cellular Metabolism
Source: J Extracell Vesicles. 2026 May 28;15(6):e70288. doi: 10.1002/jev2.70288 (PMC13239353; doi:10.1002/jev2.70288)
Supplement: Supplementary file 1 — Supporting material: jev270288‐SUP‐0001‐Figures.pdf [file JEV2-15-e70288-s001.pdf]

Supporting Information for

Title: DAMP laden extracellular vesicles from the airways of patients with severe SARS-CoV-2 respiratory infection compromise inflammation and cellular metabolism.

Short title: METABRONA

Authors: April Rees[1Ä*], Oliver Richards[1Ä], Molly E Raikes[1], Megan Chambers[1], Sophie G Reed[1], Ceri Battle[2], Hannah Toghill[2], Luke Newey[2], Stephen J Evans[1], Tyler J Joseph[1], Haiyan An[1], Jason Webber[1], Iain Perry[1], Nicholas Jones[1], and Catherine A Thornton[1]

[Ä]These authors contributed equally to this project.
[1] Institute of Life Science, Swansea University Medical School, Swansea, Wales, UK, SA2 8PP
[2] Physiotherapy, Morriston Hospital, Swansea Bay University Health Board, Swansea, Wales, UK, SA6 6NL

[*]Corresponding author:
Dr April Rees
ILS1, Swansea University Medical School
Singleton Campus
Swansea University
Swansea, Wales, UK
SA2 8PP

Telephone: 01792 987806
Email: april.rees@swansea.ac.uk

Funding: This work was funded by the Medical Research Council (MRC), Tackling COVID-19 project under grant MR/V037013/1.

Keywords: SARS-CoV-2, extracellular vesicles, monocytes, macrophages, blood, aspirates

Abbreviations: Bronchial airway fluid (BAF), mononuclear cells (MNCs), extracellular vesicles (EVs), mononuclear phagocytes (MNPs), coronavirus disease 19 (COVID-19), severe acute respiratory syndrome coronavirus 2 (SARS-CoV-2)

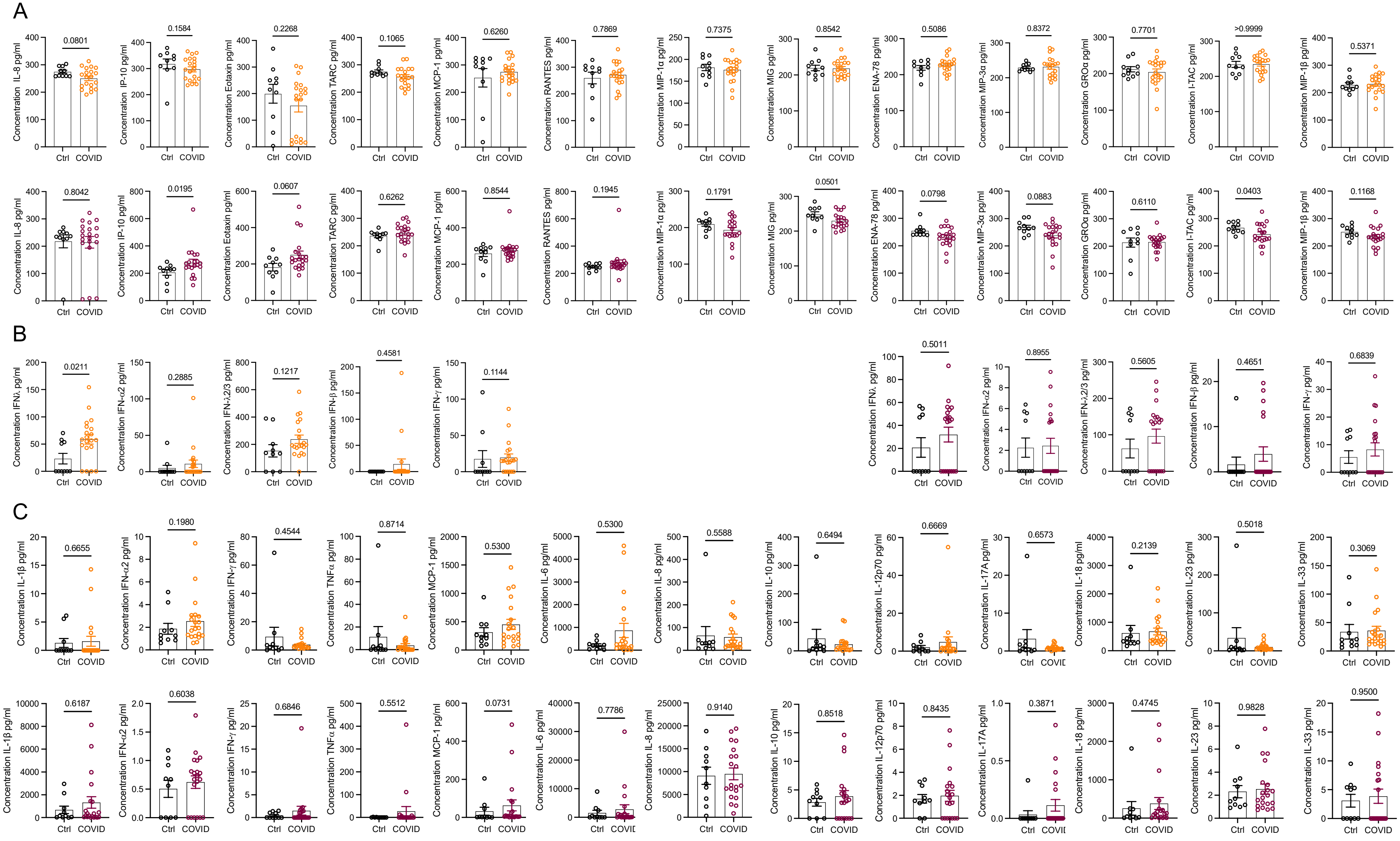

Supp Fig. 1: Bar charts with individual points of the measurement of cytokines and chemokines in blood-plasma and BAFfor control (n=10) versus COVID-19 (n=20). To determine significance, Mann-Whitney tests were used, where p <0.5 was significant. LegendPlex^{TM} panels were used to measure **(A)** proinflammatory chemokines, **(B)** interferon response, and **(C)** proinflammatory cytokines.

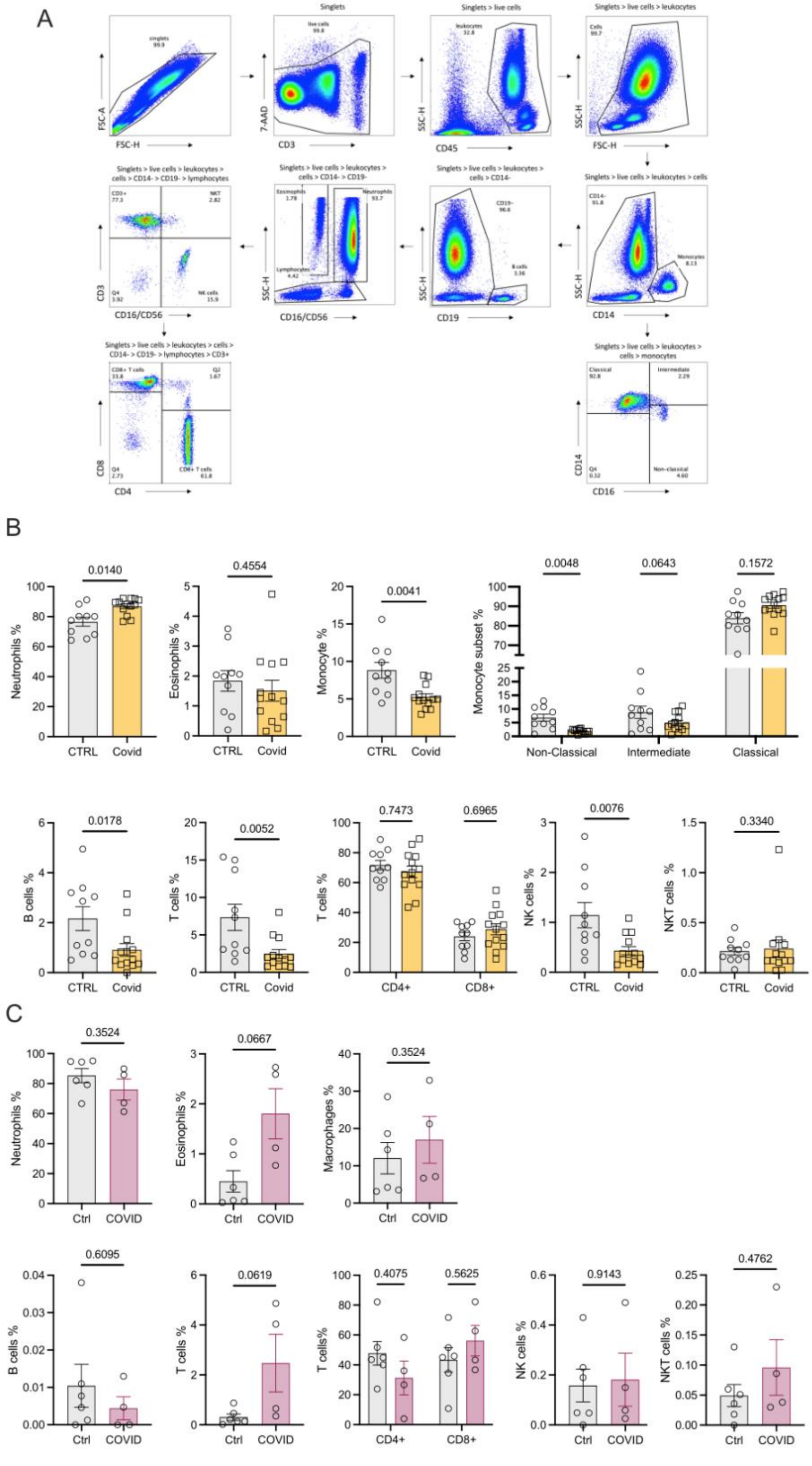

Supp Fig. 2: Flow cytometry data of blood and BAFusing the Miltenyi © 8-colour panel. **(A)** Gating strategy to identify neutrophils, eosinophils, monocytes and its subsets, B cells, T cells and its subsets, NK cells and NKT cells. Bar charts with individual points are illustrated for control versus COVID in **(B)** blood (n=10, n=13) and **(C)** BAF(n=6, n=4). Statistics were performed using either Mann-Whitney tests, or a two-way ANOVA with a Sidak's post-hoc test.

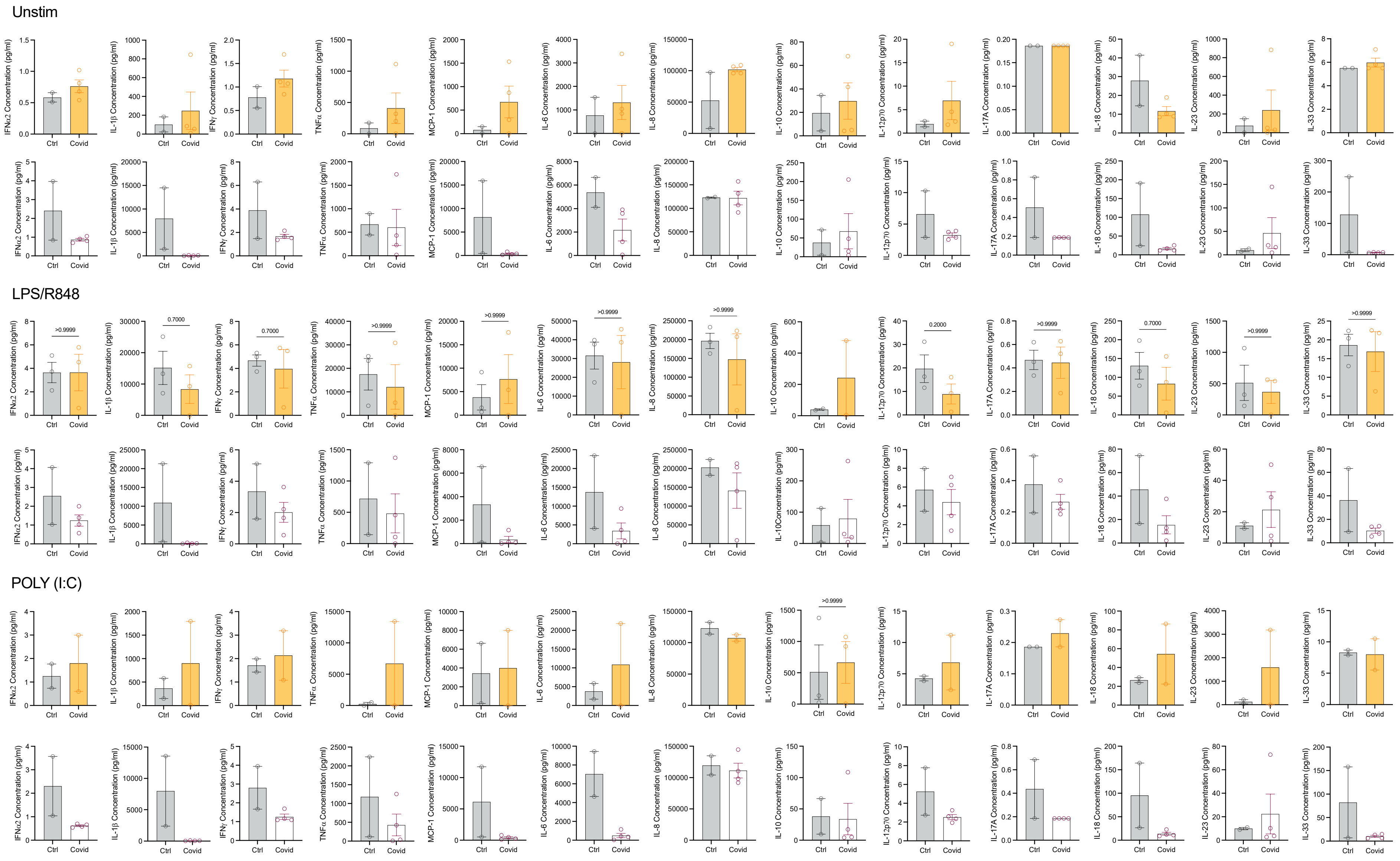

Supp Fig. 3: Bar charts with individual points of the measurement of cytokines produced by MNPs obtained from ventilated control versus COVID-19 patients. MNPs were either left unstimulated (n=2, n=4), or stimulated with LPS and R848 (blood n=3; BAFn=2, n=4), or POLY (I:C) (blood n=2; BAFn=2, n=4) for 24 hours, and supernatants archived for later measurement of cytokines. Statistics were only performed on the blood-derived MNPs for LPS and R848 due to the number of replicates; a Mann-Whitney t test was performed.

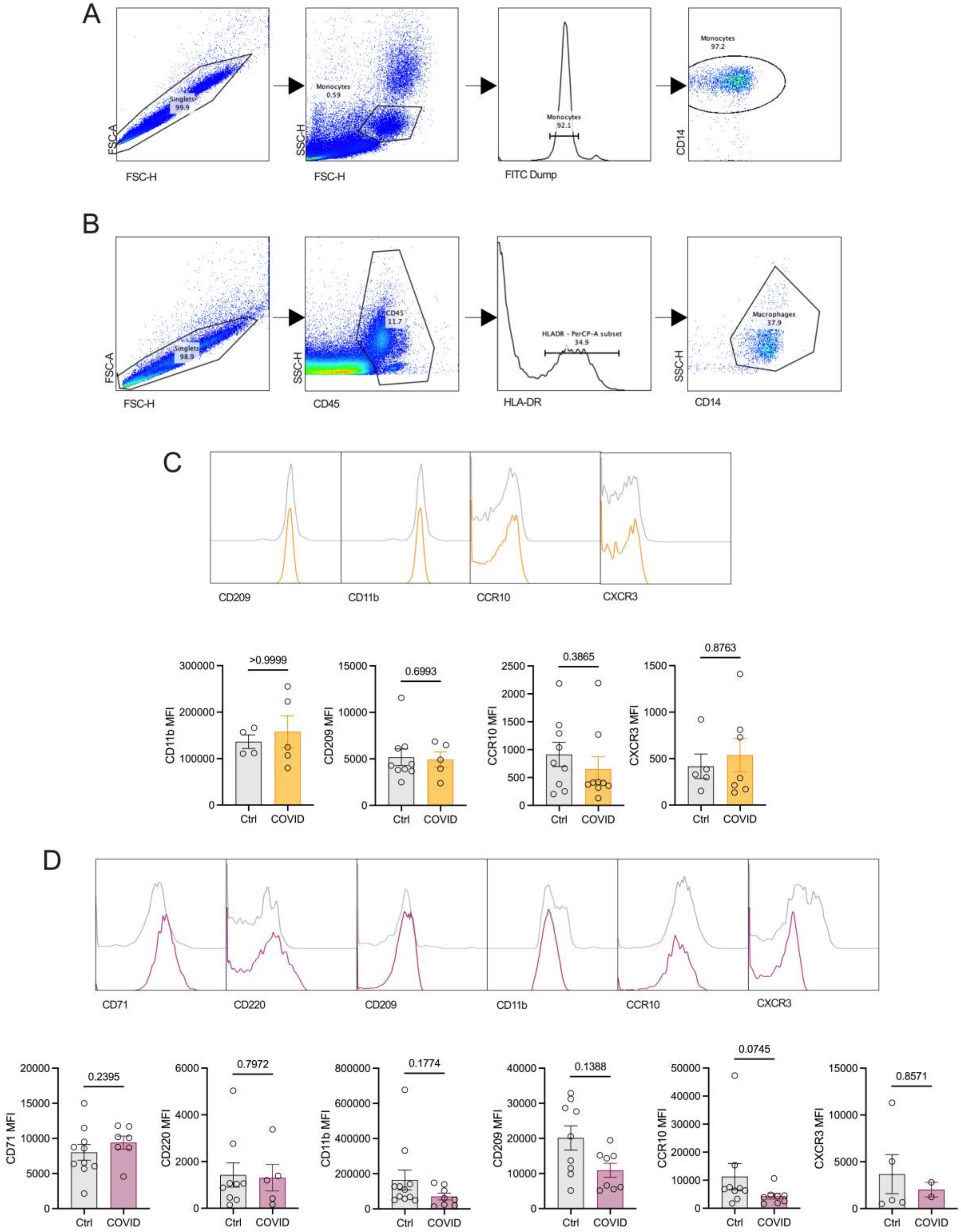

Supp Fig. 4: Flow cytometry data of blood-derived and BAF-derived MNPs. Gating strategies for **(A)** blood monocytes and **(B)** BAFmacrophages. **(C)** Histograms and individual dot plots for CD11b, CD209, CCR10, and CXCR3 for blood monocytes. **(D)** Histograms and individual dot plots for CD71, CD220, CD11b, CD209, CCR10, and CXCR3 for BAFmacrophages. To determine significance, Mann Whitney t tests were used.

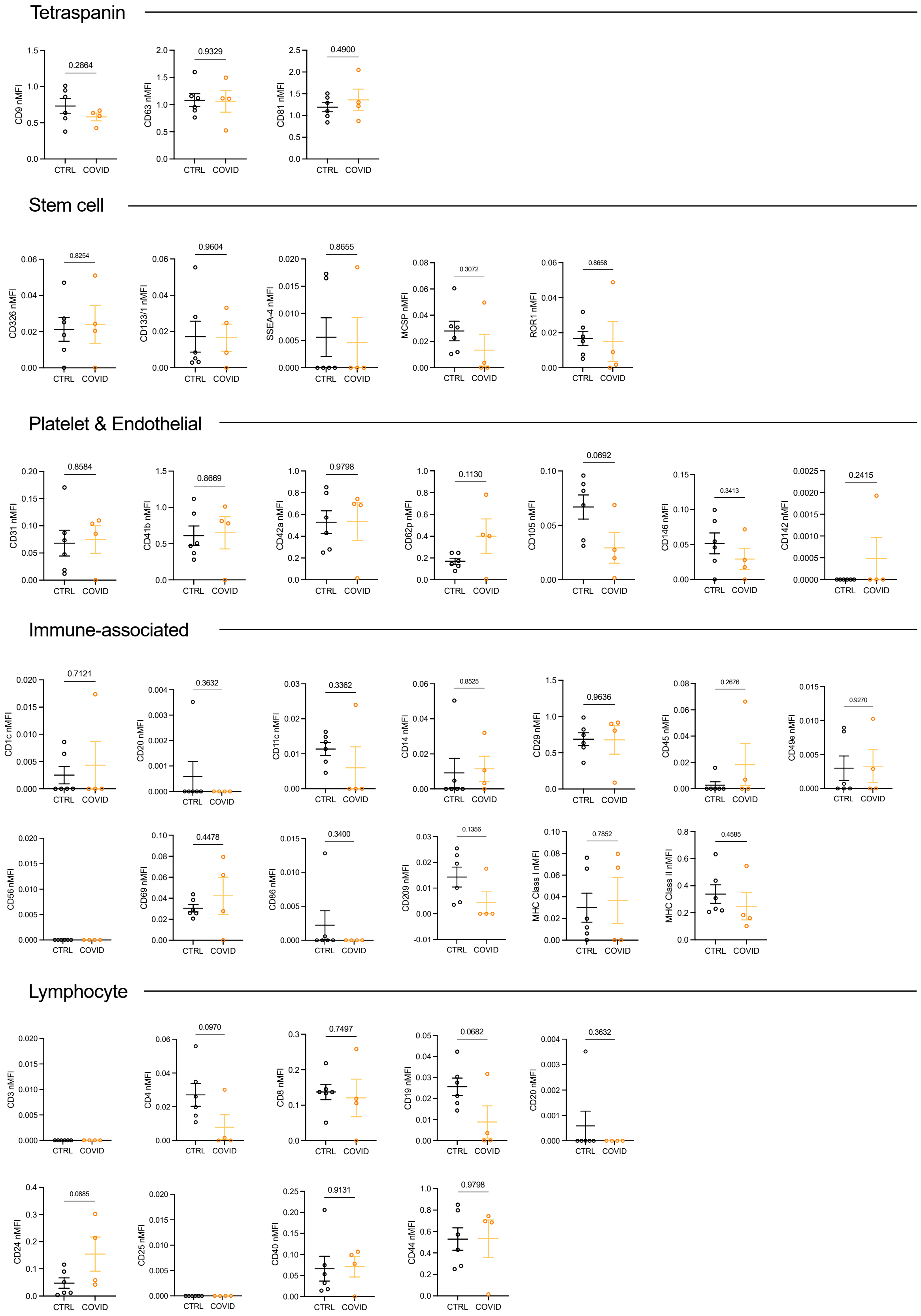

Supp Fig. 5: Graphs with the individual points of surface markers on blood-derived EVs from control (n=6) versus COVID-19 (n=4) patients. Markers were measured via MACSPlex™ and organised based on their association with cells of origin. To determine significance, Mann Whitney t tests were used.

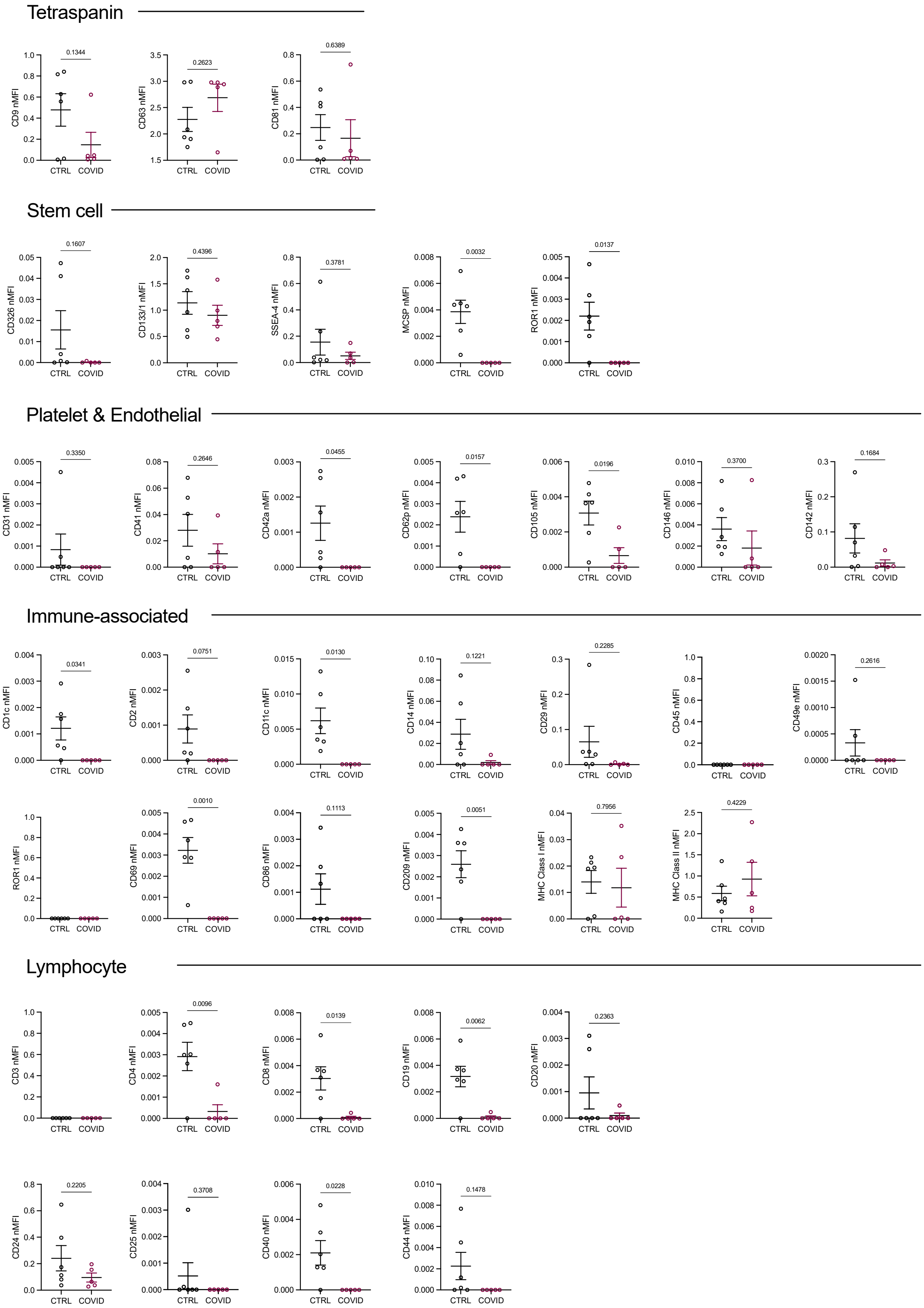

Supp Fig. 6: Graphs with the individual points of surface markers on BAF-derived EVs from control (n=6) versus COVID-19 (n=5) patients. Markers were measured via MACSPlex^{TM} and organised based on their association with cells of origin. To determine significance, Mann Whitney t tests were used.

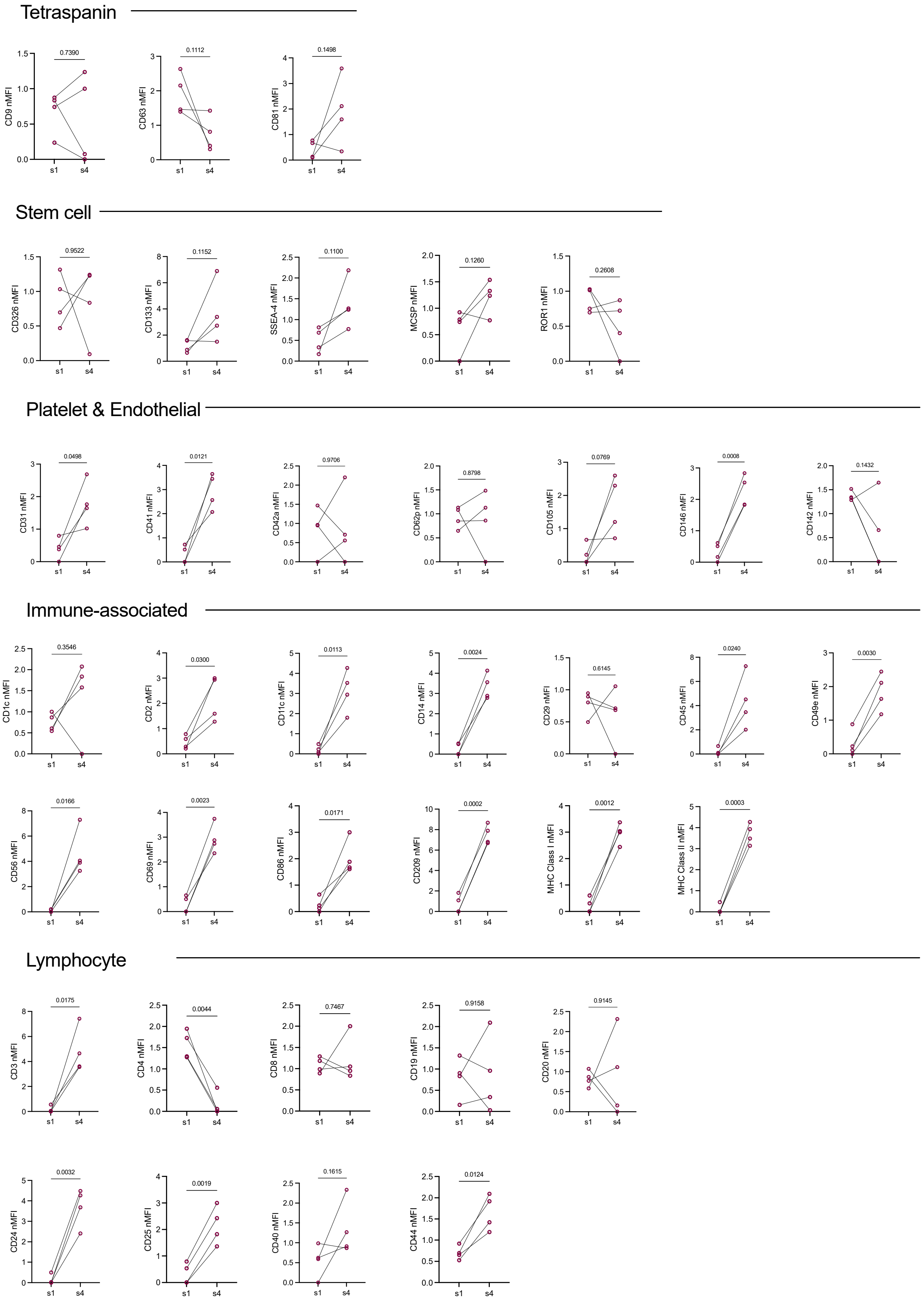

Supp Fig. 7: Graphs with the individual points of surface markers on BAF-derived EVs from matched COVID-19 patients at day 0 and day 7 who had recovered (n=4). Markers were measured via MACSPlex^{TM} and organised based on their association with cells of origin. To determine significance, Paired t tests were used.

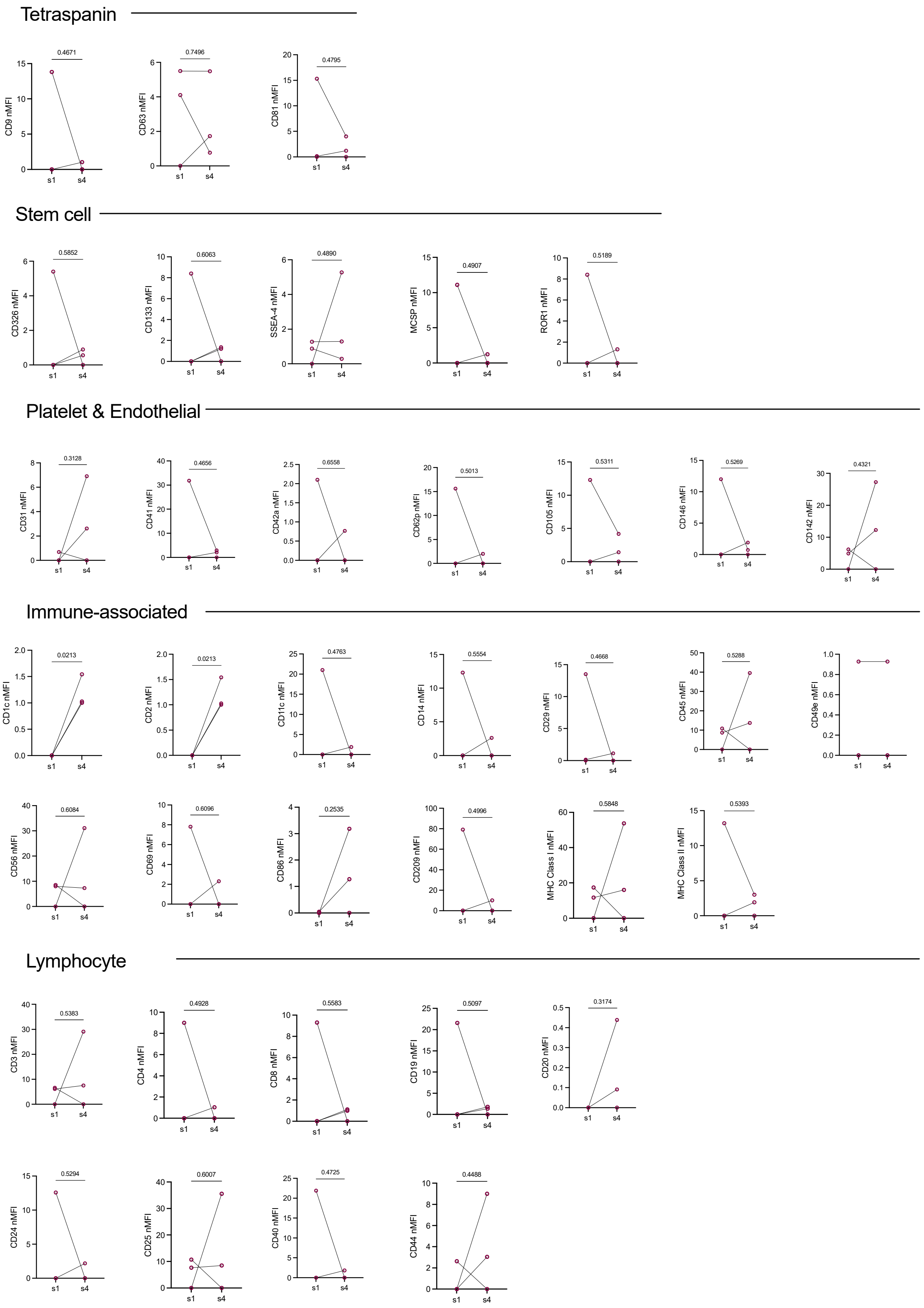

Supp Fig. 8: Graphs with the individual points of surface markers on BAF-derived EVs from matched COVID-19 patients at day 0 and day 7 who had died (n=3). Markers were measured via MACSPlex^{TM} and organised based on their association with cells of origin. To determine significance, Paired t tests were used.

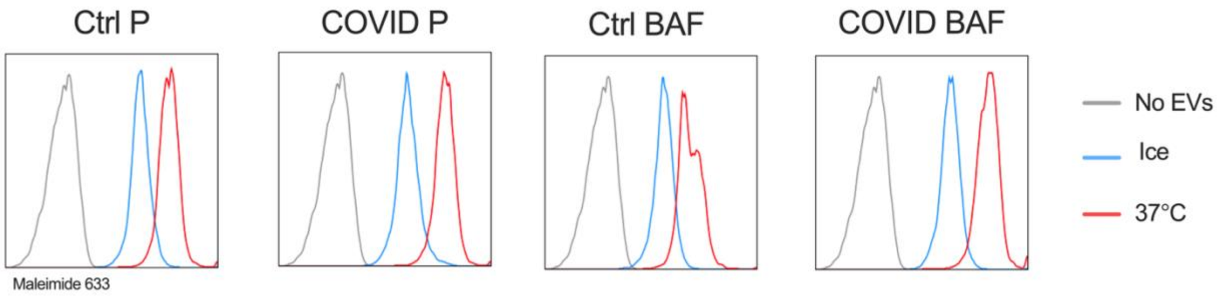

Supp Fig. 9: Histograms of EV uptake by monocytes. Monocytes were cultured without EVs (grey), or with EVs at 37C (red) or on ice (blue; negative control).

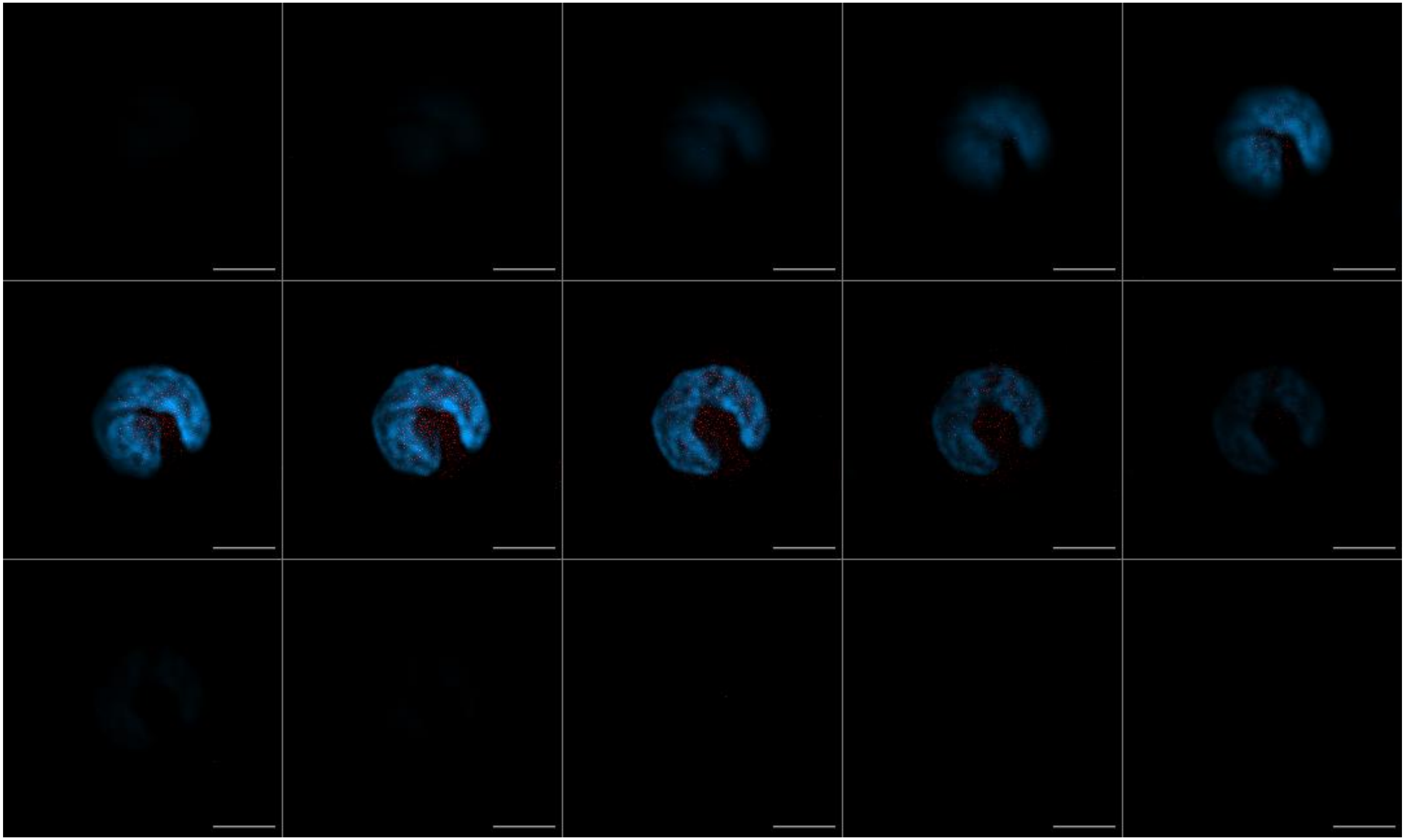

Supp Fig. 10: Full Z-stacks for plasma control EV uptake by monocytes.

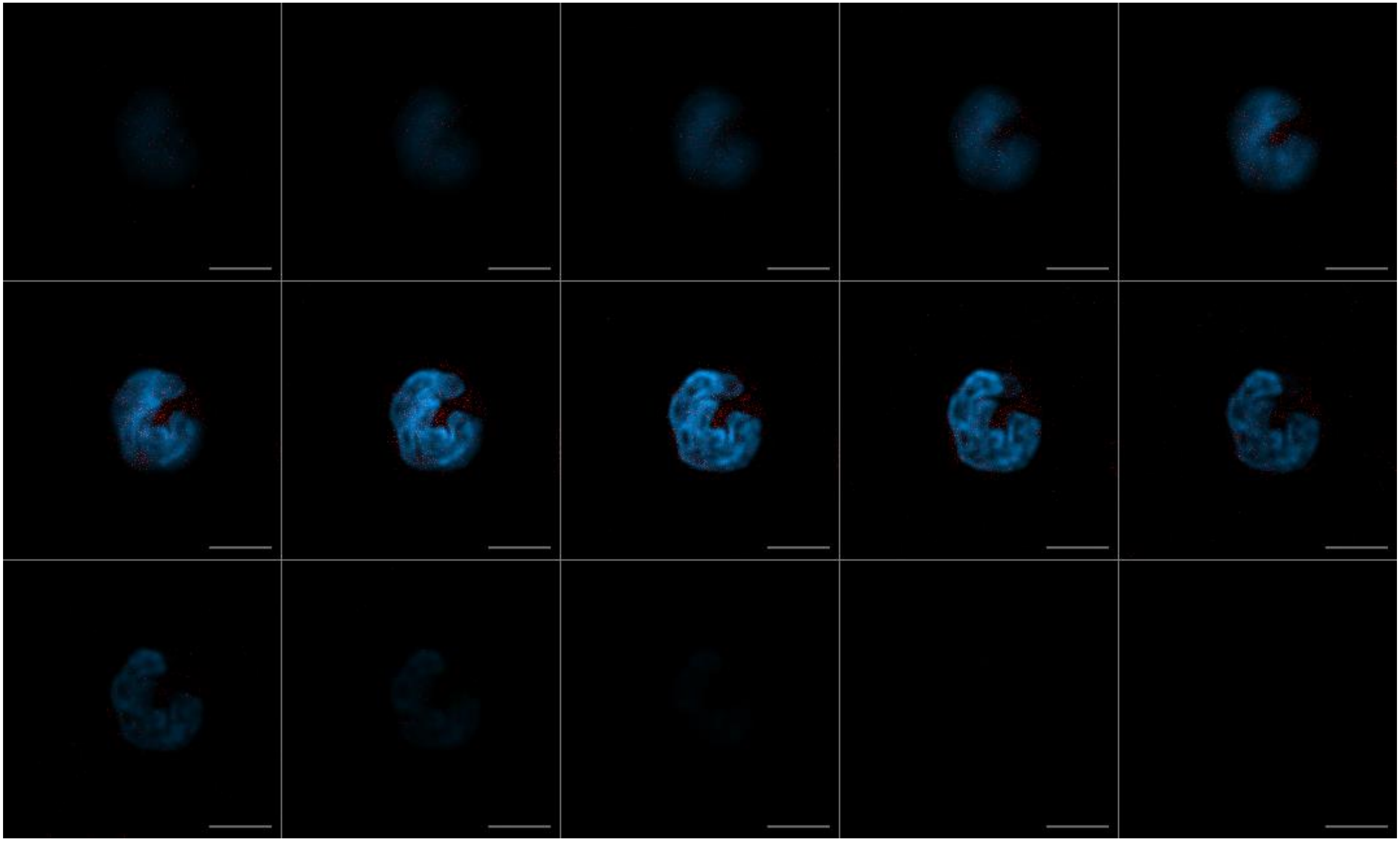

Supp Fig. 11: Full Z-stacks for plasma COVID EV uptake by monocytes.

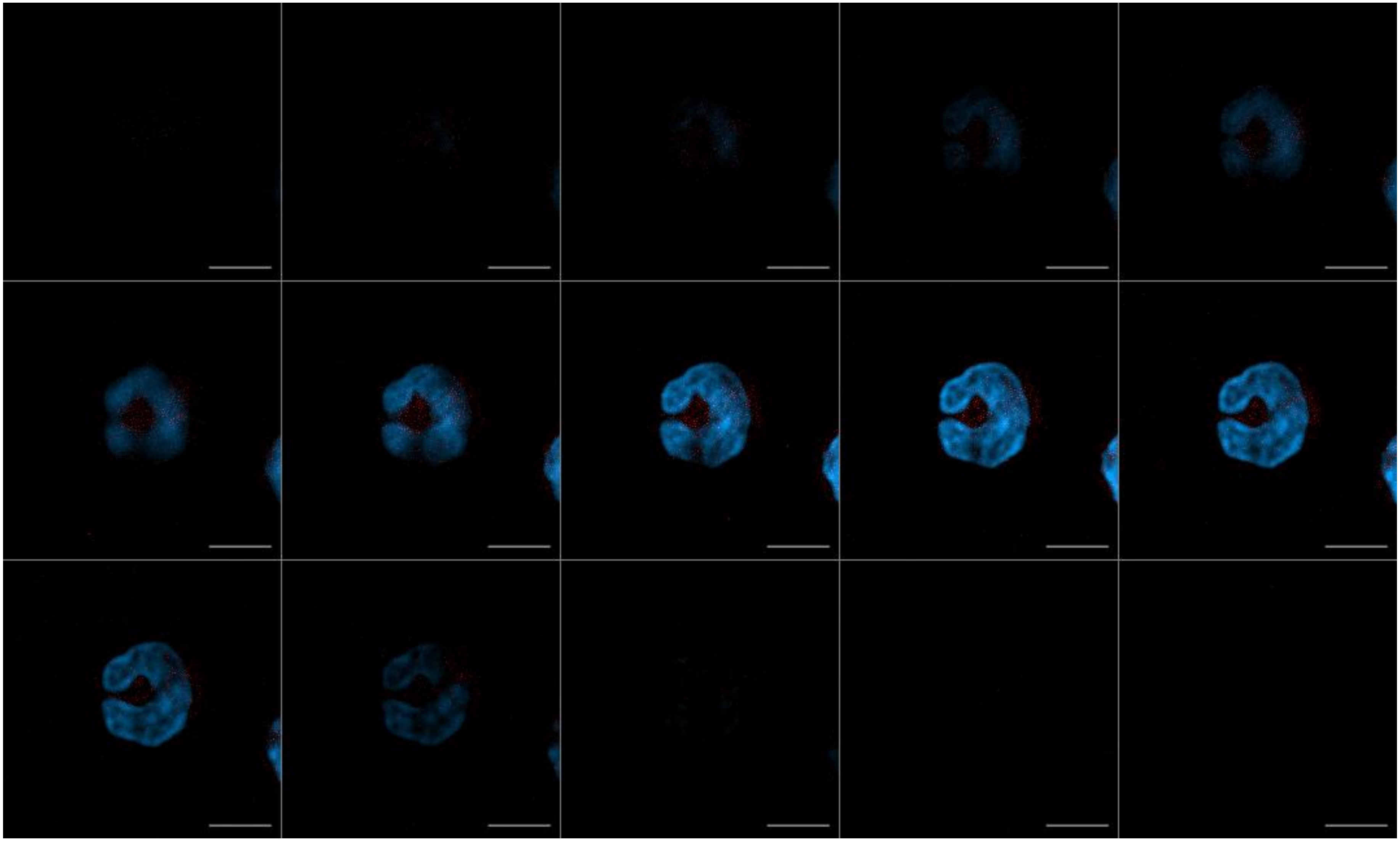

Supp Fig. 12: Full Z-stacks for aspirate control EV uptake by monocytes.

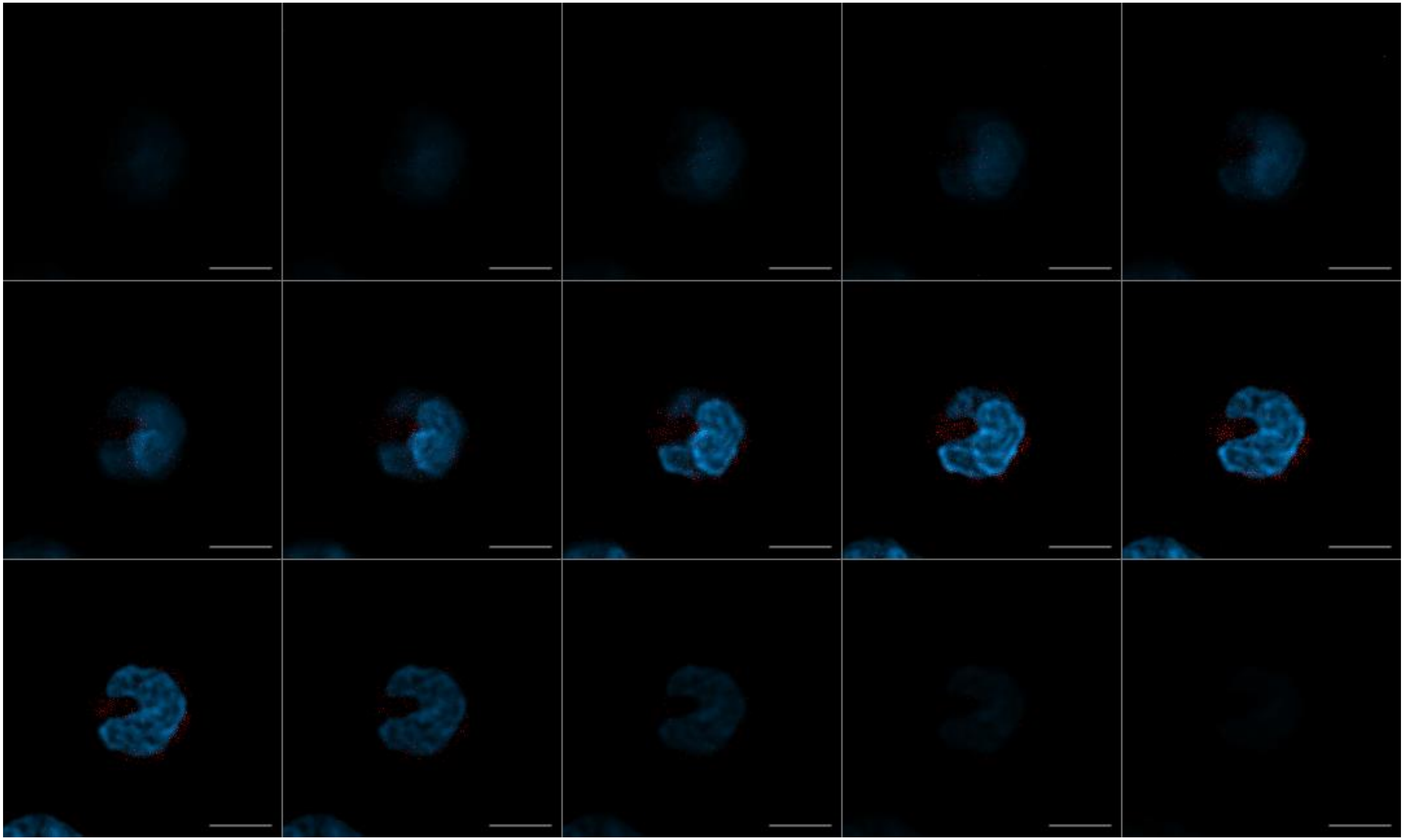

Supp Fig. 13: Full Z-stacks for aspirate COVID EV uptake by monocytes.
